# MS2Tox Machine Learning Tool for Predicting the Ecotoxicity of Unidentified Chemicals in Water by Nontarget LC-HRMS

**DOI:** 10.1021/acs.est.2c02536

**Published:** 2022-10-21

**Authors:** Pilleriin Peets, Wei-Chieh Wang, Matthew MacLeod, Magnus Breitholtz, Jonathan W. Martin, Anneli Kruve

**Affiliations:** † Department of Materials and Environmental Chemistry, Stockholm University, Svante Arrhenius Väg 16, SE-106 91 Stockholm, Sweden; ‡ Department of Environmental Science, Stockholm University, Svante Arrhenius Väg 16, SE-106 91 Stockholm, Sweden

**Keywords:** LC_50_, fragmentation spectra, contaminants, nontargeted, high-resolution mass spectrometry

## Abstract

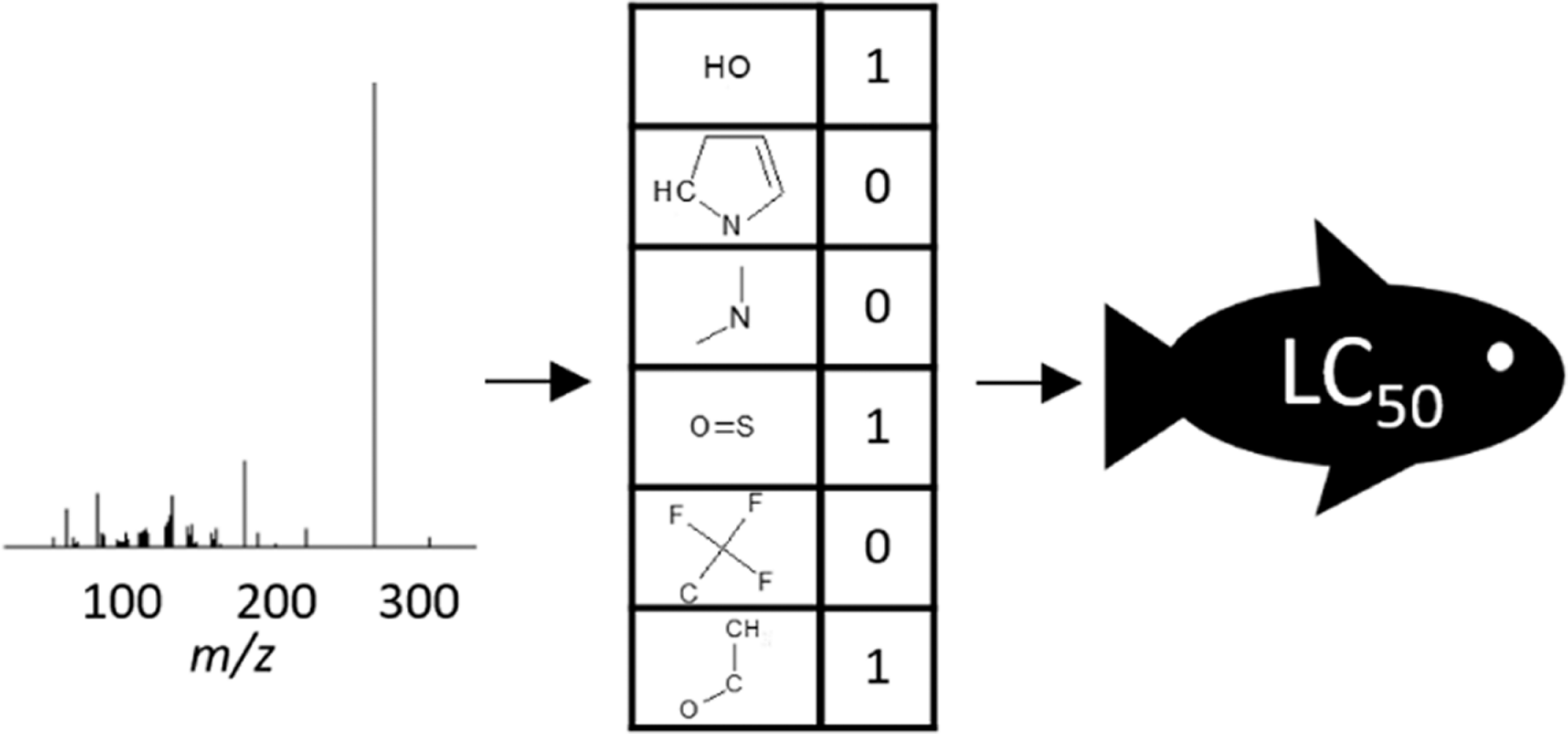

To achieve water quality objectives of the zero pollution action plan in Europe, rapid methods are needed to identify the presence of toxic substances in complex water samples. However, only a small fraction of chemicals detected with nontarget high-resolution mass spectrometry can be identified, and fewer have ecotoxicological data available. We hypothesized that ecotoxicological data could be predicted for unknown molecular features in data-rich high-resolution mass spectrometry (HRMS) spectra, thereby circumventing time-consuming steps of molecular identification and rapidly flagging molecules of potentially high toxicity in complex samples. Here, we present MS2Tox, a machine learning method, to predict the toxicity of unidentified chemicals based on high-resolution accurate mass tandem mass spectra (MS^2^). The MS2Tox model for fish toxicity was trained and tested on 647 lethal concentration (LC_50_) values from the CompTox database and validated for 219 chemicals and 420 MS^2^ spectra from MassBank. The root mean square error (RMSE) of MS2Tox predictions was below 0.89 log-mM, while the experimental repeatability of LC_50_ values in CompTox was 0.44 log-mM. MS2Tox allowed accurate prediction of fish LC_50_ values for 22 chemicals detected in water samples, and empirical evidence suggested the right directionality for another 68 chemicals. Moreover, by incorporating structural information, e.g., the presence of carbonyl-benzene, amide moieties, or hydroxyl groups, MS2Tox outperforms baseline models that use only the exact mass or log *K*_OW_.

## Abstract 2

Thousands of toxic chemicals are overlooked in targeted analysis of water samples. This work presents MS2Tox to pinpoint toxic chemicals in water samples with the aid of machine learning.

## Introduction

The quality of our water resources depends to a large extent on the inherent toxicity and concentration of chemical pollutants that are present.^1^ Modern nontarget analysis by liquid chromatography high-resolution mass spectrometry (LC-HRMS)^2^ allows rapid profiling of thousands of molecular features in drinking water,^3,4^ surface water,^5−7^ and wastewater.^8−11^ These substances are complex mixtures present in water, including anthropogenic substances (e.g., pesticides and industrial chemicals),^2,7,8,12^ natural substances (e.g., natural dissolved organic matter),^6^ endogenous human metabolites,^13^ and a multitude of associated transformation products with unknown aquatic toxicities.^4^

Identification of the molecular structure associated with each mass spectrometry feature remains a bottleneck in nontarget analyses. This process is slow and highly laborious, ultimately requiring confirmation by authentic chemical standards to reach “Level 1” confidence.^14^ Moreover, only a very small fraction of the thousands of detected features are identifiable in this manner. For example, in a nontarget analysis of household dust, only 33 chemicals could be unequivocally identified from over 5000 features,^15^ and in Swiss wastewater, only 1.2% of the features with an unequivocal chemical structure could be identified.^16^ Nevertheless, there is normally a large amount of relevant data from each feature that could potentially inform toxicological knowledge, even if the molecule cannot be unequivocally identified. This relevant data includes chromatographic retention times and mass spectral (MS) information, such as accurate mass and isotope patterns in full scan spectra (MS^1^) and data-rich structural information in associated fragmentation spectra (MS^2^).^8,17^

Even among the molecules that can be unequivocally identified by nontarget analysis, there may not be adequate aquatic toxicological information available.^9^ Whole effluent toxicity testing with relevant in vivo bioassays is the most accurate means of testing the toxicity of real-world effluents, but this method is not rapid and does not identify the chemical source(s) of toxicity without follow-up effect-directed analyses, which is furthermore expensive and highly laborious. For chemicals identified with nontarget analysis that lack experimental toxicity values, read-across methodologies and quantitative structure–activity relationship (QSAR) models can be used to predict toxicity.^18−25^ Read-across methods are based on the knowledge of common toxicophores—in other words, distinct structural features or moieties that bestow a toxic property.^26,27^ Other QSAR^28^ models may use physicochemical properties that are either measured or estimated from structure, along with functional groups present in the chemical, to predict toxicity.^22^ Application of such read-across and QSAR models has, so far, not been effectively paired with nontarget LC-HRMS analysis because of the presumed need to first have an unequivocal molecular structure.

The analytical behavior of an analyte in LC-HRMS, its physicochemical properties, and its toxicity are all connected to its molecular structure, and thus toxicity could be correlated directly with analytical data. There is a reason for optimism since retention time is correlated with log *K*_OW_:^29,30^ the accurate mass and isotope pattern of the molecular ion reveal the elemental composition, including the presence of heteroatoms and halogens,^31^ and MS^2^ spectra may indicate the presence of functional groups (e.g., −NH_2_, −OH, CO_2_H) by fragments and neutral losses.^12^ These data are sufficient to characterize many known toxicophores and are similar to structural alerts systems used in read-across^32,33^ and certain QSAR models.^34^

**Figure 1:**
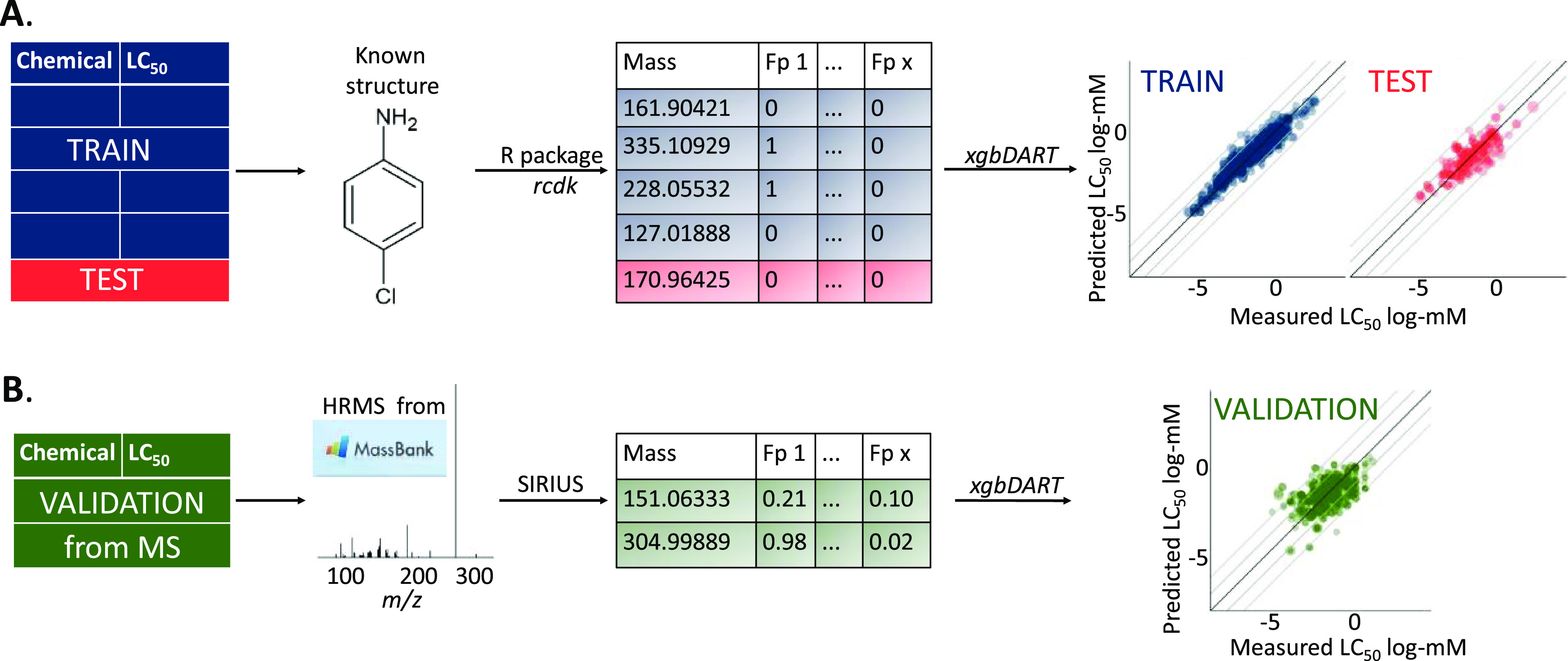
Workflow for development of the MS2Tox prediction models. Organic chemicals with toxicity values and MS^2^ spectra in available databases were used as the validation set, while chemicals with available toxicity values but no MS^2^ spectra were randomly divided into training and test sets. (A) In the initial training and testing of MS2Tox, molecular fingerprints (FPs) were calculated from chemical structure using *rcdk* and used to train a gradient-boosted prediction model using *xgbDART*. (B) In the validation stage, fingerprints were calculated from empirical HRMS spectra using SIRIUS+CSI:FingerID software, and these fingerprints were then used to predict toxicity with the *xgbDART* model that was trained in (A). The same workflow was repeated for all ecotoxicological endpoints: static fish LC_50_, flow-through fish LC_50_, water flea LC_50_, water flea EC_50_, and algae EC_50_.

Here, we test the hypotheses that analytical characteristics from typical LC-HRMS nontarget data acquisition of water samples could be used to predict the toxicity of unidentified molecules and moreover that machine learning strategies could be applied to unravel these structural dependencies. For structural characterization, fingerprints that show the presence or absence of functional groups and structural parts can be used.^35,36^ Here, we developed a method and tool to predict toxicity values from nontarget LC-HRMS data to enable hazard evaluation for unidentified organic molecules. We use the tool to predict the in vivo 50% lethal concentration (LC_50_) or effective concentration (EC_50_)^19^ in ecotoxicologically relevant test organisms. A machine learning approach combines the molecular mass and molecular fingerprints from structure for training and testing (Figure 1A) to predict LC_50_ in fish species (*Lepomis macrochirus*, *Pimephales promelas*, *Oncorhynchus mykiss* combined) and water flea (25 different clones and species combined) as well as EC_50_ predictions for water flea (8 different clones and species combined, see the Supporting Information section “Toxicity Data from CompTox”) and green algae (10 different clones and species combined, see the SI section “Toxicity Data from CompTox”). For validation, fingerprints from MS^1^ and MS^2^ (HRMS) spectra are used (Figure 1B). The resulting root mean square error (RMSE) was less than an order of magnitude in log-mM for the best fish LC_50_ model and water flea EC_50_ model, demonstrating proof of concept that toxicity can be predicted from HRMS spectra. The model could thus be a useful tool for flagging inherently toxic molecules in complex mixtures.

## Materials and Methods

### Toxicity Data

LC_50_ and EC_50_ values for fish in static and flow-through exposures and for water flea and algae were obtained from the EPA CompTox Chemicals Dashboard.^37^ For each endpoint used, the parameters are presented in SI Table S1. All concentrations for predictions were expressed as mM and converted to a logarithmic scale. For chemicals where one species had multiple independently measured LC_50_ and EC_50_ values, the median was used. The reported experimental LC_50_ and EC_50_ values for one chemical varied significantly with a pooled standard deviation of 0.25–0.44 log-mM depending on the endpoint. Chemicals with experimental standard deviation larger than 1.5 log-mM were deemed unreliable and excluded from datasets.

### Training and Testing MS2Tox

For training and testing the model, fingerprints were calculated from structure using the *get.fingerprint()* function from the R package *rcdk*.^38^ Substructure, MACCS, PubChem, Klekota–Roth, custom-made SMARTS, and ring system fingerprints were calculated. For model training, *xgbDART* from the *xgboost* library was used from the *caret* package. Prior to training, chemicals that had MS^2^ spectra in MassBank (datasets from Eawag, University of Athens, and LCSB) were filtered out and assigned to the validation set. The remaining chemicals were divided into training (80%) and test (20%) sets. For hyperparameter tuning in the training of a model, a 10-fold cross-validation was additionally used. A weight was given to the median toxicity value based on the standard deviation of the individual toxicity values such that chemicals with more precise toxicity values had a higher impact in the model training. Chemicals with only one measured value were given weight corresponding to the average standard deviation. For analyzing how much each fingerprint affected the model, *varImp()* and *dummyVars()* functions from *caret* were used. For variable importance, SHapley Additive exPlanations (SHAP) graph of the first 10 variables using *shap.score.rank()* from GitHub package Shap visualization for xgboost was used.^39^ For comparing predicted and measured toxicity values, *R*^2^, *Q*^2^, and RMSE were used.

### Validation

MS^2^ data from MassBank^40^ measured by Eawag, LCSB, and the University of Athens were used for validation of MS2Tox. The datasets were selected so that a reasonable proportion of data was left for training, testing, and validation. For example, for the fish static LC_50_ dataset, the amounts were 517, 130, and 219, respectively. MS^2^ spectra that had identical LC-HRMS measurement parameters but different collision energies were written into one .ms file for fingerprint calculations. This was done since SIRIUS+CSI:FingerID software uses MS^2^ data to build fragmentation trees, and adding spectra measured on lower and higher collision voltages together gives more characteristic fragmentation trees (SI section “HRMS Data from MassBank” for detailed explanation). Due to the lack of MS^1^ spectra in MassBank, the isotope pattern was calculated from the chemical formula. Multiple fingerprint predictions were obtained for one chemical if both negative and positive mode spectra were available or by different research groups or with different LC-HRMS parameters. For validation, fingerprints were calculated using SIRIUS+CSI:FingerID version 4.9.5.^41,42^ All of the chosen parameters for SIRIUS+CSI:FingerID are described in the SI section “Fingerprints Calculated with SIRIUS Software”.

### Final Model

For the final application, structural fingerprints were also calculated for chemicals in the validation set, and all training, test, and validation set data were used for model training. The function *FishLC50Prediction()* for predicting fish static and flow-through LC_50_ values is available in R package *MS2Tox* on GitHub (https://github.com/kruvelab/MS2Tox).

### Application Measurements

For testing the final model, in-house HRMS acquisitions were used. Three solutions containing 152 unique spiked chemicals were analyzed by a Thermo Scientific Dionex Ultimate 3000 (Thermo Fisher Scientific) with an RS binary pump and Thermo Scientific Q Executive Orbitrap. Solvents were acetonitrile and MilliQ water with 0.1% formic acid (pH = 2.7), and separation was carried out on a reversed-phase column Kinetex 2.6 μm EVO C18 (150 × 3.0 mm^2^) from Phenomenex in gradient elution mode. For the acquisition of MS^2^ data, an inclusion list with the theoretical exact mass of all of the spiked chemicals was used. Detailed instrumental parameters and a list of all of the spiked chemicals are given in the SI section “Application Solutions”.

## Results and Discussion

**Figure 2:**
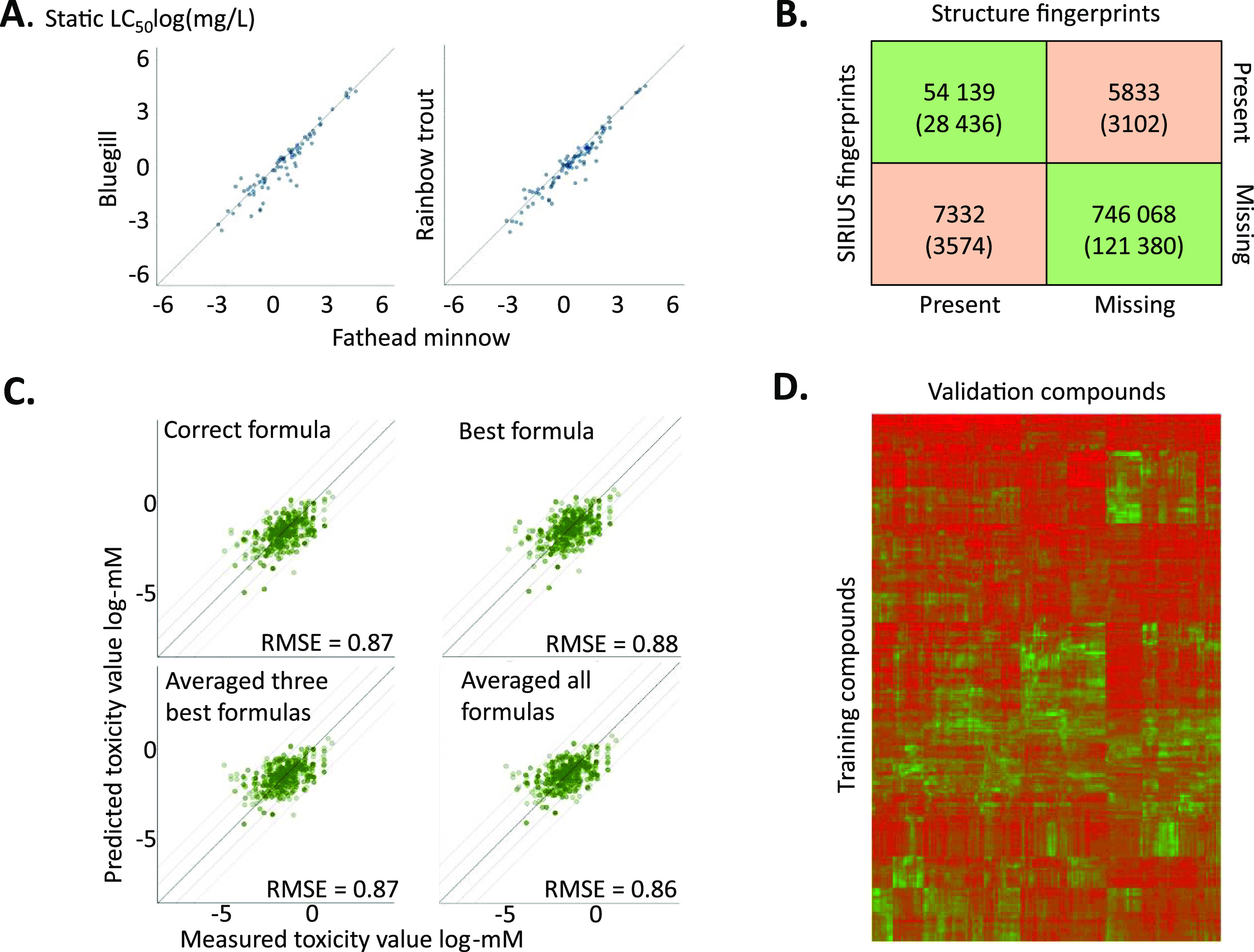
Data selection and processing steps. (A) Correlation of LC_50_ values for bluegill and rainbow trout with fathead minnow. The black line shows an ideal agreement of the LC_50_ values for the two fish species, indicating equal sensitivity. (B) Cross table of the fingerprints calculated from the SMILES of the chemical and fingerprints predicted by fragmentation trees and support vector machines from SIRIUS+CSI:FingerID software. The number on top represents all calculated fingerprints, while the number below in parentheses is the number of these fingerprints that were actually used by the final machine *xgbDART* model for predicting fish static LC_50_ values. (C) Agreement between measured and predicted toxicities depending on the assigned molecular formulas by fragmentation trees. (D) Heat map of the cosine similarity of fingerprints for chemicals in training and validation sets. The similarity ranged from zero (red) to one (green), and the color gradient is shown in 10 equal steps. The abundance of green areas for each column in the graphs shows that for a chemical in the validation set, a highly similar chemical exists in the training set; here, we see abundant green areas for a majority of the chemicals.

Machine learning algorithms work best when they are trained with large sets of accurate data. Therefore, publicly available toxicity data in CompTox^37^ were screened to evaluate the size and quality of available data. The number of experimentally measured toxicity values available for model training was smaller than that for chemical parameters (e.g., log *K*_OW_, p*K*_a_), but promising datasets suitable for training machine learning models were flow-through LC_50_ values for fathead minnow and water flea (640 and 387 unique chemicals, respectively) and EC_50_ values for water flea and algae (730 and 353 unique chemicals, respectively). Data cleaning and preprocessing revealed that LC_50_ values for fathead minnow were highly correlated with LC_50_ values for rainbow trout and bluegill, considering 91 and 74 chemicals for which LC_50_ values with both species were available (Figure 2A). Assuming that the mode of action for these species is similar, we combined the LC_50_ values into a common “fish” dataset using a general additive model (GAM).^43^ Using the GAM allowed converting the toxicity values available for bluegill and rainbow trout to the scale of fathead minnow, whereby a Gaussian regression fitted over common chemicals accounts for sensitivity differences between species. As a result, five datasets were obtained after data processing: fish static LC_50_, fish flow-through LC_50_, water flea LC_50_, water flea EC_50_, and algae EC_50_, having 871, 841, 379, 728, and 353 unique chemicals, respectively.

### Model Training and Testing

To train the model for predicting toxicity from the HRMS spectra (MS^2^ and MS^1^), the HRMS spectra need to be converted into information relevant to the presence or absence of endpoint-related toxicophores. The structural fingerprints calculated from HRMS spectra^42^ yield a probability of the presence of specific structural moieties and are promising as a means to flag the presence of toxicophores; however, they have not yet been tested for such a purpose. Training a machine learning model directly from the experimental HRMS spectra and toxicity values is impractical due to a modest intersection of chemicals with publicly available experimental toxicity endpoints and MS^2^ spectra. To overcome this hurdle, we first trained a machine learning model based on the molecular fingerprints calculated from the structure of all of the chemicals for which experimental toxicity values were available; to allow predictions for unknown chemicals later in the validation stages, we only used the molecular fingerprints that could also be deduced from HRMS spectra (Figure 1). Thus, the dataset available for model training was limited only by the experimental toxicity values; to train the machine learning models, we considered fingerprints that (1) could be calculated with fragmentation trees and support vector machine (SVM) in SIRIUS+CSI:FingerID^42^ from HRMS spectra and with the *rcdk* package^38^ from structure and (2) could be calculated both in negative and positive ionization modes. Altogether 1263 fingerprints for each chemical were calculated and used alongside the exact mass. Thereafter, highly correlated fingerprints and fingerprints with near-zero variance were removed, and approximately 200 fingerprints remained for model training, depending on the organism dataset.

**Figure 3:**
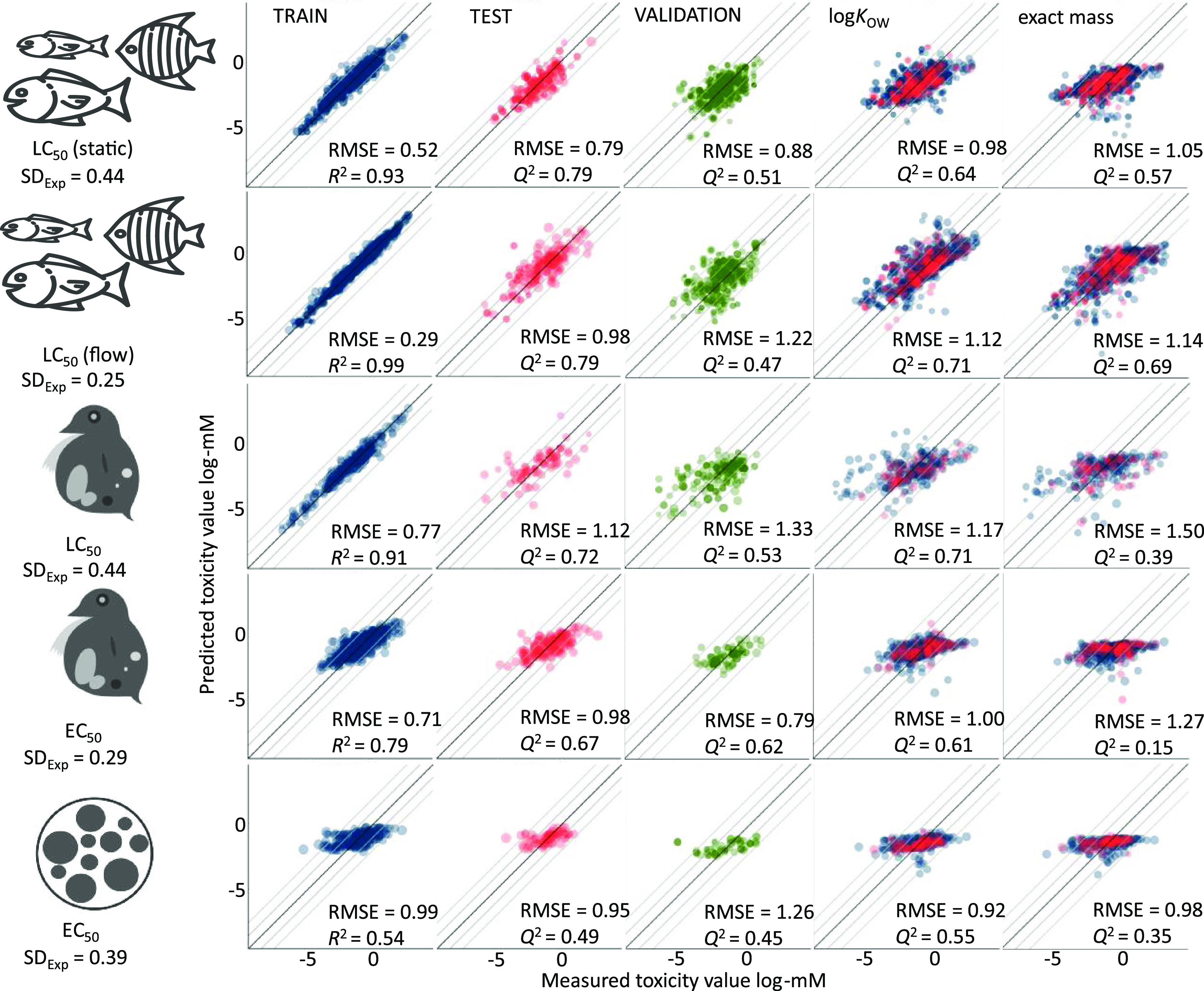
Overview of the performance of MS2Tox models for training, test, and validation sets for fish (rows 1 and 2), water flea (rows 3 and 4), and algae (row 5). For comparison, prediction models trained only with log *K*_OW_ and exact mass using linear regression are visualized on the right. Blue dots in the graph represent the training set, pink dots represent the test set, and green dots represent the validation set. SD_Exp_ is the experimental standard deviation from logarithmic endpoint concentrations retrieved from CompTox. The root mean square error (RMSE) shows the difference between experimental and predicted toxicity values, while *R*^2^ and *Q*^2^ evaluate the correlation. The darker middle line on the graph shows an ideal case where predicted toxicity values agree with the experimental values. Lighter lines mark the difference of 1 and 2 log-mM from ideal prediction. The first two rows show the result for the final models that are represented in the MS2Tox package.

Various regression models, such as SVM, random forest regression, and different gradient boosting algorithms, were trained with the *caret* package in R.^44^ The trained models were compared based on root mean squared error (RMSE) and squared correlation coefficients (*R*^2^ and *Q*^2^) observed from independent training and test sets (see SI). The extreme gradient boosting Dropouts Additive Regression Trees (*xgbDART*)^45^ method was chosen as the best-performing algorithm. Altogether, five different prediction models were trained: static and flow-through LC_50_ models for fish, LC_50_ and EC_50_ models for water flea, and an EC_50_ model for algae. For all five models, the RMSE for the test set was close to 1.0 log-mM unit, with values ranging from 0.79 to 1.12 log-mM (see the TEST column in Figure 3). With the exception of the LC_50_ (flow-through) model, RMSE values for the training set were less than a factor of two lower than those for the test set, indicating that the models did not suffer from excessive overtraining (Figure 3).

### Toxicity Predictions from Mass Spectra

Available mass spectral datasets in MassBank^40^ from Eawag, University of Athens, and LCSB (University of Luxembourg) were used to validate the toxicity predictions from MS^2^ spectra. All chemicals used for validation had not been used in the training and test set, and thus the validation performance indicates the performance under near real-world conditions where the chemicals of interest are unknown. Models predicting static LC_50_ values for fish and EC_50_ values for water flea yield mean prediction error below 1.0 log-mM; however, for the LC_50_ model of water flea, an RMSE of more than 1.1 log-mM was observed (Figure 3). The results in positive and negative ionization modes did not differ significantly. For the fish static LC_50_ model, the RMSE for negative mode (0.85 log-mM) was slightly lower than that for positive mode (0.90 log-mM) despite the fact that the positive mode dataset was three times larger than negative mode dataset. The RMSE for the static fish LC_50_ model (0.88 log-mM) was only twice as large as the standard deviation of experimental values (0.44 log-mM) for the same dataset, which is small in relation to the wide range of toxicities, from −6.3 to 2.9 log-mM. The best-performing model was the static LC_50_ model for fish, wherein 75% of the predicted toxicities differed by less than one log-mM unit from the experimental values, and correspondingly 98% of predicted toxicities differed by less than two log-mM units. The highest error was for permethrin, which was predicted to be ∼1300× less toxic than its corresponding experimental LC_50_ value. In this specific case, the wrong formula was assigned, and thus incorrect fingerprints, by fragmentation trees and SVM in SIRIUS+CSI:FingerID, which explains the incorrect toxicity. The correct formula of permethrin ranked second, and using this, the predicted toxicity was only 6× less toxic than the experimental LC_50_. For the salicylanilide spectra from the LCSB dataset, LC_50_ was also underpredicted by 1000×, even though the correct formula was assigned by SIRIUS+CSI:FingerID with 98% of correctly calculated fingerprints from all fingerprints.

The prediction errors of MS2Tox depended on a number of factors: (1) the accuracy of the fingerprints predicted with fragmentation trees and SVM; (2) the similarity of chemicals of interest and the chemicals used for model training; and (3) accuracy of the *xgbDART* machine learning model in predicting toxicity from the fingerprints. The molecular fingerprints calculated from the fragmentation trees may have inaccuracies due to a low number of fragments observed in some MS^2^ spectra. The number of structurally meaningful fragments in MS^2^ spectra depends on molecular structure and the collision energy, sensitivity, scanning range, and mass resolution of the instrument.^46,47^ Over all datasets, 98.4% of the 813,372 fingerprints calculated with SIRIUS+CSI:FingerID were correctly predicted. Here, correct predictions mean that if a specific fingerprint was present in the structure, its predicted probability was above 0.5 as the xgbDART treats all values below 0.5 as zeros and values above 0.5 as ones (Figure 2B). For positive ionization mode spectra, the results were slightly better than those for the negative mode: 98.6% correct fingerprints compared to 97.6%, respectively. Nevertheless, in the two worst cases, almost 200 of 1263 possible fingerprints were incorrectly calculated for the chemical (2*R*,6*S*)-fenpropimorph based on two MS^2^ spectra measured by two different research groups, Athens University and Eawag. In both cases, the fragmentation spectra seemed to contain a sufficient number of peaks (22 selected by SIRIUS+CSI:FingerID). Some of the most incorrectly calculated fingerprints belonged to amide and carbonyl groups (SI Table S8).

Insufficiently characteristic MS^2^ spectra may ambiguously correspond to multiple fragmentation trees and, therefore, different structural fingerprints. Moreover, for some spectra, more than 10 molecular formulas were predicted. If the fingerprint probabilities differ significantly for alternative molecular formulas, machine learning will also predict different toxicity values for each molecular formula. The predicted molecular formulas are ranked based on plausibility by SIRIUS+CSI:FingerID (i.e., “SiriusScore”); however, correct formulas may not always be ranked highest. It was of interest to see the impact of the assigned molecular formula on the probabilities of molecular fingerprints and therefore the LC_50_ values. For this reason, three different approaches were evaluated here: (1) toxicity values were predicted for all obtained molecular formulas and averaged; (2) toxicity values were predicted for three highest-ranked molecular formulas and averaged; and (3) toxicity was predicted only for the molecular formula ranked highest. For all cases, the RMSEs were between 0.86 and 0.88 log-mM with no significant difference. As results with the highest-ranked molecular formula and with the correct molecular formula were most similar (Figure 2C), the fingerprints corresponding to the highest-ranked molecular formula were used for final toxicity predictions. This indicates that even if the correct formula is not ranked the highest, the fingerprints can still be predicted accurately. Histogram (SI Figure S3) and explanation showing good cosine similarities between fingerprints from the same MS^2^ data but different assigned formulas are given in the SI section “Fingerprints Calculated with SIRIUS Software”.

The model prediction accuracy was also influenced by the similarity of the “unknown chemicals” in the validation set relative to chemicals used for model training. The structural similarity of the chemicals in the validation set and the training test set was evaluated based on the cosine similarity of calculated fingerprints and principal component analysis (see Figure S2 in SI). Heat map analysis (Figure 2D) showed that 70% of validation chemicals had at least 20 training set chemicals with the cosine similarity exceeding 0.5 for fish static LC_50_. The good similarity observed justifies using the chosen fingerprints and training set chemicals for training the MS2Tox model with application on chemicals detectable with LC-HRMS. On the other hand, the model accuracy may also be affected by the similarity of the clones and species combined into one dataset for the water flea and algae model. Here, 25, 8, and 10 species and clones were combined for the water flea LC_50_, water flea EC_50_, and algae EC_50_ model, respectively, and the heterogeneity of species included may have significantly worsened the learning power of the machine learning models. Unfortunately, no single clone or species has a sufficient number of measured toxicity values available for training a model.

### Model Interpretation

**Figure 4:**
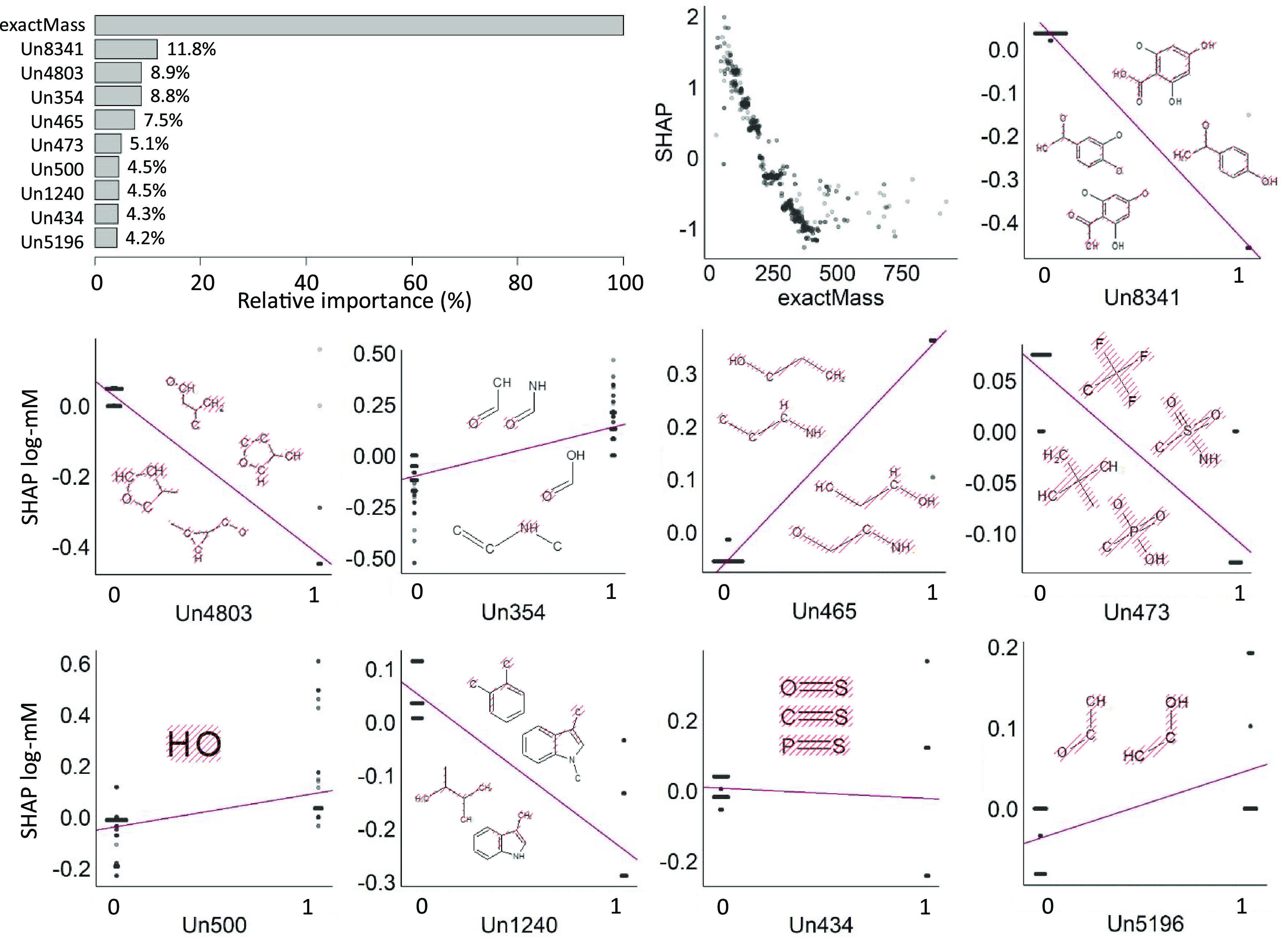
Variable importance for the 10 most important variables in the fish static LC_50_ model. The bar chart in the top left corner shows the variable importance relative to the most important variable, exact mass. The associated SHAP graphs of each variable show the magnitude and directionality of each variable on the predicted LC_50_. For each of the molecular fingerprints, the *x*-axis indicates the absence (0) or presence (1) of the respective structural fragment, and lower SHAP values indicate lower predicted LC_50_ values (higher toxicity), assuming all other parameters are constant. The line shows the directionality of the impact of the descriptors. Fingerprint naming “Un” refers to the absolute index numbering system in SIRIUS+CSI:FingerID. Graphical descriptions from SIRIUS+CSI:FingerID^42^ software is also shown for each fingerprint SHAP plot.

To understand the information obtained from the *xgbDART* algorithm, the importance and contribution of each fingerprint to the prediction was evaluated with SHapley Additive exPlanations (SHAP). For the fish static LC_50_ MS2Tox model trained on the dataset extracted from CompTox, the most important descriptor, by far, was the molecular mass, with the relative importance of the next variable (carbonyl-benzene moiety) being only 12% of the former. From the SHAP plot in Figure 4, it can be seen that the predicted LC_50_ value drops by almost 3 log-mM when the molecular mass increases from 26 to 400; however, a further increase in molecular mass does not decrease the predicted LC_50_ values. The high importance of the molecular mass variable is not surprising given that (1) it is the only continuous variable in the dataset and (2) increasing molecular mass is known to be correlated with increased acute toxicity,^48^ since the baseline mechanism of acute toxicity is narcosis, i.e., nonspecific reversible disturbance of membrane functioning.^49^

Seventeen fingerprints had relative importance over 2% as large as the importance of molecular mass in the fish static LC_50_ model. The low relative importance of fingerprints (maximum 11.8% as large as molecular mass) indicates that the information about specific functional groups and elements present in the molecule fine-tunes the toxicity predictions. The most important fingerprints for fine-tuning the fish static LC_50_ model were the presence of a carbonyl-benzene moiety, the presence of a ketone, amide, or hydroxyl group. A detailed investigation of SHAP (Figure 4) indicated that an increase in molecular mass alongside the presence of aromatic carboxyl acids, ketone groups, and double-bonded sulfur increased the toxicity of the chemicals. For example, the addition of aromatic carboxyl acids decreased the LC_50_ value on average by 0.4 log-mM.

Given the high variable importance and SHAP values of molecular mass, we evaluated the extent to which structural fingerprints added predictive power to the machine learning models and thus may allow the prediction of specific modes of toxicity in addition to narcosis. To assess this, a simplified linear model with only exact mass was compared with the *xgbDART* model using exact mass together with fingerprints. The RMSE decreased with fingerprint addition, suggesting that a machine learning model could be feasibly used to predict specific modes of toxicity beyond narcosis. To probe this evidence further and to evaluate if the trained models are able to predict more than just hydrophobicity-driven narcosis, a linear model between log *K*_OW_ values retrieved from CompTox and toxicity values was investigated.^50^ It was observed that predictions based only on log *K*_OW_ yielded higher errors in both the high- and low-toxicity regions. The RMSE for the log *K*_OW_ test model was 0.98 log-mM, compared to 0.79 log-mM for the main model. Moreover, a poor correlation compared to the machine learning model (*R*^2^ 0.57 for the training set vs 0.93, and *Q*^2^ 0.64 for the test set vs 0.79 (Figure 3)) was observed when comparing predicted and measured toxicity values using log *K*_OW_.

### Application to Spiked Aqueous Solution

**Figure 5:**
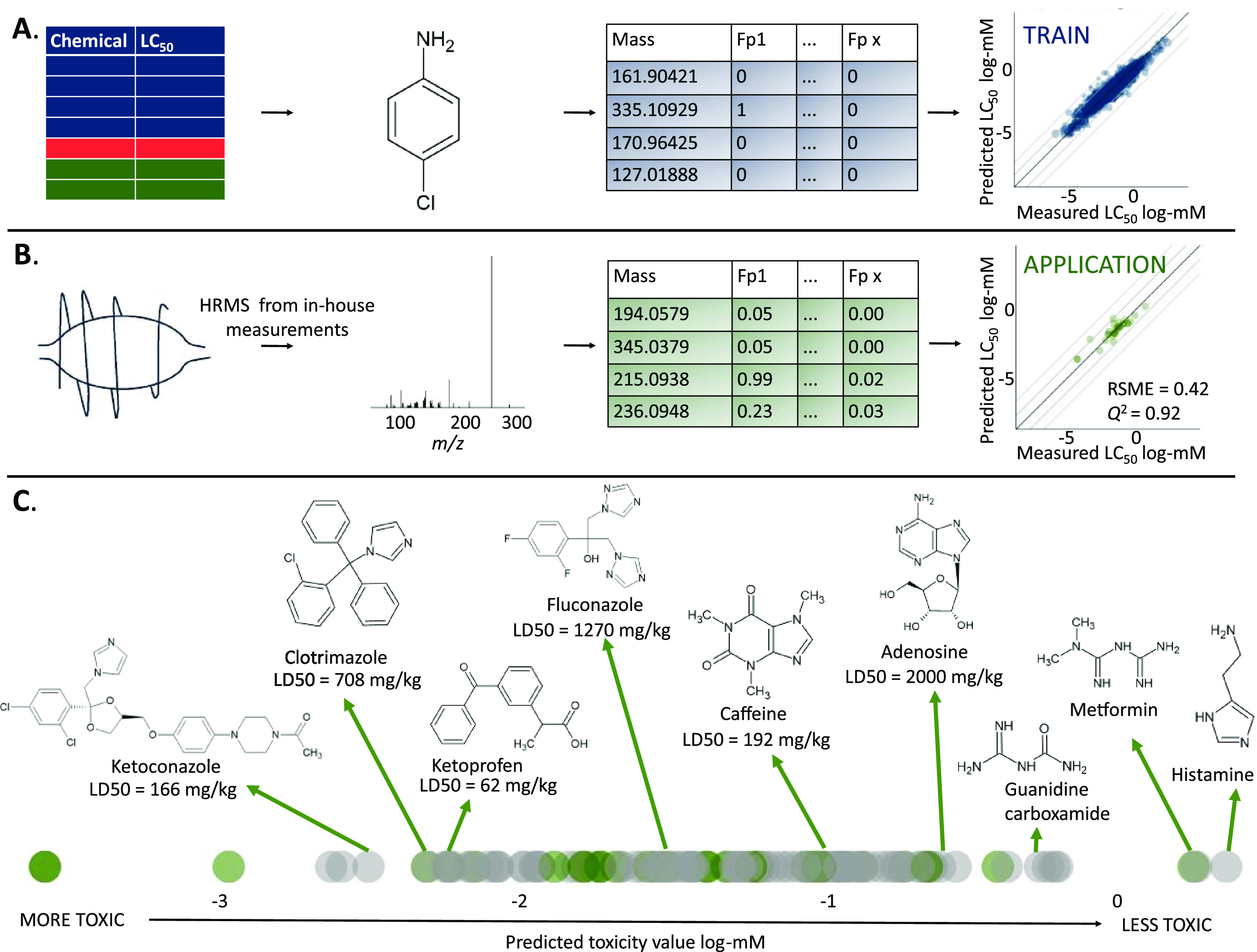
Final model training of the fish static LC_50_ prediction model and application to authentic HRMS MS^2^ spectral data from water sample analyses. (A) Toxicity values from training, test, and validation sets were compiled into a larger training set, and the model was retrained based on the structural fingerprints. (B) From in-house measured HRMS (Orbitrap) spectra, fingerprints were calculated using SIRIUS+CSI:FingerID software, and toxicity was predicted with the final trained model. Chemicals with known toxicity were compared. (C) Green data points are MS2Tox-predicted toxicity values with a corresponding experimental value in the database (last graph in part (B)). Chemicals that did not have fish static LC_50_ value are represented as gray data points, and thus validation of these points is not possible. Transparent points are used to show in which regions more overlapping points are present. Structures, names, and rat LD_50_ values are given below for some of the chemicals that did not have LC_50_ values for fish as an indicative validation.

The MS2Tox model for the prediction of fish static LC_50_ was applied to evaluate the potential for toxicity predictions of unknown substances. We used three spiked water samples (see SI Tables S4–S6) distributed in a NORMAN interlaboratory comparison and previously analyzed in-house by a data-dependent MS^2^ LC-HRMS acquisition on an Orbitrap instrument. For the LC_50_ predictions, the MS2Tox model was retrained so that all available LC_50_ values were included in the model training to yield a model most suitable for practical application. For 90 unique chemicals, 121 HRMS data-dependent MS^2^ spectra were recorded in negative and positive electrospray ionization mode and treated as belonging to unidentified chemicals. For 22 of the chemicals, experimental LC_50_ values from CompTox were available and were compared with the predicted values, yielding an RMSE below 0.5 log-mM. For 68 chemicals, no experimental fish static LC_50_ values were available for comparison. However, even for these chemicals, the plausibility of the predictions can be assessed against expectations based on toxicity tests in other organisms and the identity of the substances. For some of the chemicals, the lethal dose 50% (LD_50_) values for rats were available in CompTox.^37^ Although LC_50_ values for fish and LD_50_ for rats may show significant differences due to different uptake pathways, a comparison between the two effect values could still be used to classify substances as high, medium, and low toxicity. For example, chemicals like ketoprofen had relatively high predicted toxicity (i.e., low LC_50_) for fish and similarly high experimental toxicity for rats. Substances on the least-toxic end of the predicted spectrum were chemicals including histamine, guanidine carboxamide, and adenosine (Figure 5). Although corresponding LD_50_ values for these latter chemicals are unavailable, histamine and adenosine are endogenous in living organisms and are assumed to have low toxicity relative to other substances in the dataset.^51,52^ These results suggested that the developed model has strong potential for application to nontarget water sample analysis.

### Limitations and Future Perspectives

In this work, a machine learning-based approach, which we call MS2Tox, was developed for predicting toxicity values of unidentified chemicals from HRMS data in nontarget LC-HRMS analysis in both positive and negative modes. Given that generating new toxicity values in the laboratory can be time-consuming and expensive, MS2Tox relied on existing datasets of LC_50_ and EC_50_ values for fish, water flea, and algae from CompTox. Nevertheless, when it comes to the selection of chemicals and coverage of chemical space, MS2Tox is dependent on previous laboratory toxicology studies, which may not be representative of the wide coverage of natural and anthropogenic chemicals detectable with nontarget LC-HRMS. Due to the limited availability of such toxicity data and a likely bias in the existing database toward toxic substances, some of the relations between toxicity and mass spectrometry variables may also be biased.

The RMSE of predicted toxicity values for validation sets range from 0.79 to 1.33 log-mM depending on the species and endpoint. Fish LC_50_ yielded a higher prediction accuracy compared to the other tested endpoints. As for the EC_50_ of algae and water flea, the relationship between toxicity value and structure was ambiguous; therefore, the MS2Tox package includes the prediction of fish LC_50_ only.

Additionally, MS2Tox uses the structural fingerprints predicted from MS^2^ spectra with SIRIUS+CSI:FingerID software, and the accuracy of MS2Tox is therefore related to the accuracy of these fingerprint predictions. In the case of poor MS^2^ data (low number of fragment peaks, noisy spectrum), incorrectly calculated fingerprints can strongly affect the predicted toxicity.

In the future, we envision that it will become possible to advance MS2Tox for evaluating the toxic effects of complex mixtures, such as those present in drinking water or wastewater, as well as the contributing effect of individual compounds in these samples. This rapid knowledge will be essential for identifying toxic substances and mitigating environmental harm in real-world scenarios, such as for effluents. No additional measurements by nontarget LC-HRMS should be required, and laboratories analyzing water samples with LC-HRMS can use MS2Tox without additional equipment for toxicity testing or pure test compounds. Together with concentration predictions,^53^ MS2Tox might be used to evaluate the hazard of different waters as well as prioritize chemicals for identification and removal.

## Supporting Information Available

More detailed description of datasets, measurement parameters, and standards used; additional figures for other trained models (PDF)
