# Supplementary material for: MS2Tox Machine Learning Tool for Predicting the Ecotoxicity of Unidentified Chemicals in Water by Nontarget LC-HRMS
Source: Environ Sci Technol. 2022 Oct 21;56(22):15508–17. doi: 10.1021/acs.est.2c02536 (PMC9670854; doi:10.1021/acs.est.2c02536)
Supplement: Supplementary file 1 — es2c02536_si_001.pdf [file es2c02536_si_001.pdf]

Supplementary information for

MS2Tox machine learning tool for predicting ecotoxicity of unidentified chemicals in water by nontarget LC-HRMS

Pilleriin Peets[1], Wei-Chieh Wang[1], Matthew MacLeod[2], Magnus Breitholtz[2], Jonathan W. Martin[2], Anneli Kruve[1,2*]

[1] Department of Materials and Environmental Chemistry, Stockholm University, Svante Arrhenius Väg 16, SE-106 91, Stockholm, Sweden

[2] Department of Environmental Science, Stockholm University, Svante Arrhenius Väg 16, SE-106 91 Stockholm, Sweden

* Corresponding author anneli.kruve@su.se

Contents

Datasets 2

Toxicity data from CompTox 2

HRMS data from MassBank 4

Application solutions 7

Fingerprints calculated from the structure 16

Fingerprints calculated with SIRIUS software 18

Performance evaluation for the fingerprint calculation 20

Prediction model 22

Comparison of different machine learning models 22

Validation with MS^2 data from MassBank 25

Variable importance and SHAP analysis 25

Application interpretation 30

Datasets

For training and testing MS2Tox, the toxicity values were downloaded from EPA CompTox Chemical Dashboard (https://comptox.epa.gov/dashboard/). The HRMS data for validation was downloaded from MassBank (massbank.eu/MassBank/). In-house measurements were used for final application testing.

Toxicity data from CompTox

As toxicity values LC_{50} values for three fish (fathead minnow, bluegill, rainbow trout) and water flea and EC_{50} values for water flea and algae were used. Toxicity values were extracted so that all the parameters for one training model were the same. In CompTox some toxicity values were presented as lower than (<) or higher than (>) and those data points were not included. The parameters of the specific endpoints used in this study are shown in Table S1. Chemicals that had several different values due to replicate measurements were averaged (*median()* from *stats* package) and experimental standard deviation (SD) was calculated additionally with R function *sd()* from *stats* package. Chemicals with SD higher than 1.5 log-mM unit were excluded. Chemicals that did not have replicate measurements are marked with "NA". Before model training, all the averaged values were calculated into molar concentration using the exact mass and the final used unit for toxicity values was log-mM. Toxicity scale for training was from -6.38 to 2.94 log-mM and the exact ranges for all training, test and, validation set are in Table S2. All used averaged toxicity values with standard deviations can be found in GitHub, in file https://github.com/kruvelab/MS2Tox/blob/main/Data/FinalModelData/final_model_toxdata.txt.

Table S1. Parameters that were used to select suitable toxicity data from CompTox for each used endpoint

**Model**	**Fish Static**	**Fish Flow**	**LC_{50} water flea**	**EC_{50} water flea**	**EC_{50} algae**
**risk_assessment_class**	mortality: acute	mortality: acute	mortality: acute	short-term	ecotoxicity invertebrate
**toxval_type**	LC_{50}	LC_{50}	LC_{50}	EC_{50}	EC_{50}
**toxval_units**	mg/L	mg/L	mg/L	mg/L	mg/L
**study_type**	Mortality	Mortality	Mortality	Static	Static
**study_duration_class**	Acute	Acute	Acute	-	-
**study_duration_value**	4	4	2	1	72
**study_duration_units**	Day	Day	Day	Day	Hour
**exposure_route**	Static	Flow-through	Static	-	-
**species_common**	bluegill+ rainbow trout+ fathead minnow	fathead minnow+ rainbow trout+ bluegill		water flea	green algae

Table S2. Comparison of toxicity value ranges for training, test and validation set

	**Training range log-mM**	**Test range log-mM**	**Validation range log-mM**
**Static fish LC_{50}**	-6.26 to 2.93	-5.46 to 2.45	-4.91 to 1.10
**Static flow-through LC_{50}**	-6.38 to 2.94	-5.84 to 2.21	-5.13 to 1.23
**Water flea LC_{50}**	-7.00 to 2.82	-5.12 to 2.30	-6.15 to 0.81
**Water flea EC_{50}**	-4.01 to 2.25	-4.73 to 2.84	-3.47 to 1.15
**Algae EC_{50}**	-5.36 to 2.25	-4.29 to 0.96	-4.85 to 0.78

For the fish model toxicity values for three fish were combined using a generalized additive model (*gam()* from R package *mgcv*) to bring toxicity values for all three fish into a common fathead minnow scale. In Table S3 a summary of the final dataset: number of chemicals, pooled experimental standard deviation, and standard deviation quantiles are given. Standard deviation for an endpoint is pooled root mean square (RMS) with the equation:

$$Pooled\ RMS = \sqrt{\frac{\sum_{Chemical=1}^{N}(n_{Chemical} - 1)\sigma^2}{\sum_{Chemical=1}^{N} n_{Chemical} - N}}$$

Table S3. Summary statistics of the experimental toxicity data from CompTox for endpoints corresponding to the experimental parameters in Table S1 and averaging values for one compound

**Model**	**Fish Static**	**Fish Flow**	**LC_{50} water flea**	**EC_{50} water flea**	**EC_{50} algae**
Unique chemicals	864	841	387	730	353
Pooled RMS	0.44	0.25	0.44	0.29	0.39
Quantile 25% SD*	0	0	0	0	0
Quantile 75% SD*	0.22	0.01	0.26	0.11	0.25

* for this table chemicals that had only one toxicity value error was set as 0.

Green algae and water flea data contained several species with the same English name but different Latin name. All this data that had same English name was combined into one dataset. Therefore, species and clones for water flea LC_{50} were: *Daphnia magna, Ceriodaphnia silvestrii, Daphnia carinata, Macrothrix flabelligera, Ceriodaphnia dubia, Moina macrocopa, Daphnia pulex, Moina sp, Daphnia sp., Daphnia ambigua, Simocephalus vetulus, Daphnia longispina, Daphnia cucullata, Ceriodaphnia reticulata, Alonella sp., Chydorus sphaericus, Simocephalus elizabethae, Daphnia galeata, Daphnia laevis, Ceriodaphnia cornuta, Diaphanosoma brachyurum, Ceriodaphnia rigaudi, Moinodaphnia macleayi, Daphnia hyalina, Daphnia pulicaria*. Species and clones for water flea EC_{50} were: *Daphnia magna, Daphnia galeata, Ceriodaphnia dubia, Daphnia obtusa, Daphnia cucullata, Daphnia pulex, Daphnia sp., Daphnia magna straus*. Species and clones for green algae EC_{50} were: *Desmodesmus subspicatus, Scenedesmus capricornutum, Ankistrodesmus bibraianus, Chlorella pyrenoidosa, Chlamydomonas reinhardtii, Pseudokirchneriella subcapitata, Scenedesmus sp., Scenedesmus subspicatus, Chlorella vulgaris, Desmodesmus subspicatus chodat sag.*

HRMS data from MassBank

From MassBank three datasets by Eawag, Athens University and University of Luxembourg (LCSB) were used. Eawag and Athens University data was from version 2020.03 (https://github.com/MassBank/MassBank-data/releases/tag/2020.03) and LCSB from version 2020.10 (https://github.com/MassBank/MassBank-data/releases/tag/2020.10). For Eawag data both Eawag and Eawag_Additional_Specs datasets were used. Initial dataset sizes were 11199 spectra for Eawag, 4911 spectra for Athens University, and 6325 spectra for LCSB. Although MassBank dataset also provides chromatographic information like retention time

for some chemicals, this information was not included in the model. Retention times are affected by separation mode (reversed-phase, HILIC, ion exchange), mobile phase (pH, organic modifier, buffer type), column length, flow rate, pump type, brand, etc. For the MassBank data from the three labs 13 different chromatographic conditions were used, where the single largest dataset measured under the same chromatographic conditions consisted of 691 unique chemicals. This number is lower than what we were able to use by only using molecular fingerprints that can be computed from the MS^2 data. Though chromatographic information could be very beneficial for predicting toxicity, the data sparsity needs to be overcome first. One possibility would be to use a GAM model to convert retention times from one instrument/column/lab to another. However, this is much less likely to succeed as the column and mobile phase, especially pH do not affect all chemicals to an equal extent.[1] Also, the model would only be applicable to one separation mode, e.g. if reversed chromatography retention times are used, the predictions can also be made only for reversed-phase chromatography and not for HILIC. For that reason adding retention time information to the model would limit its later application of it.

Similarly, CCS values that can be measured with ion mobility provide information about the 3D structure of the chemicals and therefore possibly about the toxic interaction of the chemical with a protein. Unfortunately, CCS values can be obtained currently only with a very small fraction of HRMS instruments used for non-target analysis. Therefore, the training of such models as well as application would be limited.

The current version of MS2Tox allows toxicity predictions for any chromatography type, with or without ion mobility, and even for measurements where no chromatographic or ion mobility separation has been used, e.g. flow injections, paper spray, or MALDI.

For HRMS data, some of the chemicals had several measurements done with different parameters and both in positive and negative modes. Also, different collision energies were used. MS^2 spectra with otherwise identical LC-HRMS conditions but different collision energies and or resolution were written into one .ms file so that each file contained information about the collision voltage used as well as all fragments and their intensities were listed under the specific collision voltage. Combining multiple collision voltages captures more fragments, allows compiling a deeper fragmentation tree, and therefore, more accurate fingerprint predictions. For spectra obtained in ramp mode, no combining was done. For this reason, MassBank id that is seen in the final datasets corresponds to the last spectra with the same LC-HRMS parameters. An example of such a .ms file is given in Figure S1. E.g. acetochlor from Eawag dataset had fourteen spectra (EA010401-EA010414) with different collision energies and/or resolutions and for this compound, one data point with id EA010414 was created for fingerprint calculations. For fingerprint calculations, the number of unique chemicals was 824 for Athens, 913 for Eawag, and 849 for LCSB. For fingerprint calculations, only chemicals with available toxicity value in CompTox were used.

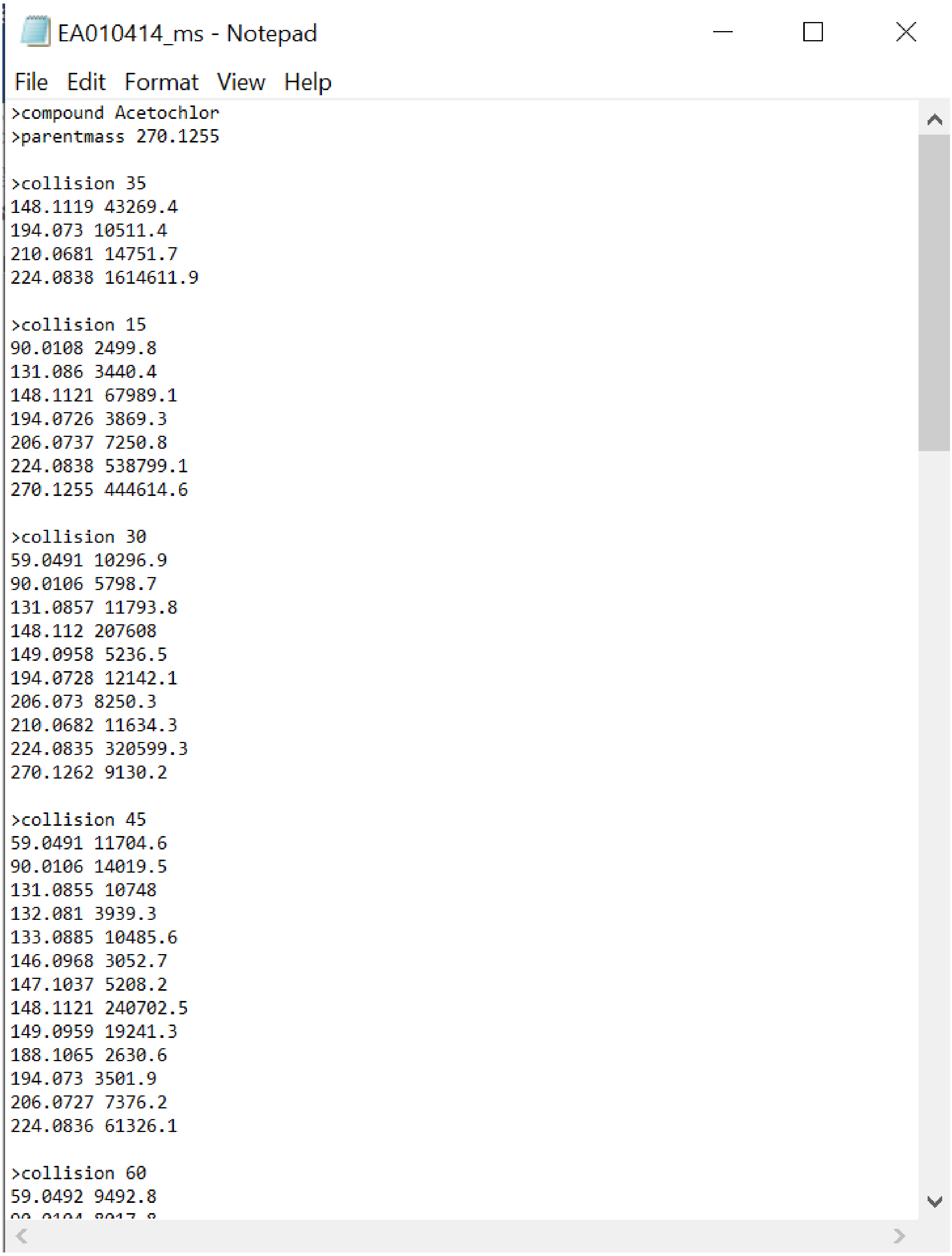

EA010414_ms - Notepad

File Edit Format View Help

```
>compound Acetochlor
>parentmass 270.1255

>collision 35
148.1119 43269.4
194.073 10511.4
210.0681 14751.7
224.0838 1614611.9

>collision 15
90.0108 2499.8
131.086 3440.4
148.1121 67989.1
194.0726 3869.3
206.0737 7250.8
224.0838 538799.1
270.1255 444614.6

>collision 30
59.0491 10296.9
90.0106 5798.7
131.0857 11793.8
148.112 207608
149.0958 5236.5
194.0728 12142.1
206.073 8250.3
210.0682 11634.3
224.0835 320599.3
270.1262 9130.2

>collision 45
59.0491 11704.6
90.0106 14019.5
131.0855 10748
132.081 3939.3
133.0885 10485.6
146.0968 3052.7
147.1037 5208.2
148.1121 240702.5
149.0959 19241.3
188.1065 2630.6
194.073 3501.9
206.0727 7376.2
224.0836 61326.1

>collision 60
59.0492 9492.8
[illegible]
```

Figure S1. Example .ms file of collected MS^2 data on different collision voltages from MassBank.

Application solutions

For application, three spiked water samples (Table S4 to Table S6), containing altogether 152 chemicals, from NORMAN inter-laboratory comparison were measured in-house. Measurements were carried out on ESI-LC-HRMS consisting of Thermo Scientific Dionex Ultimate 3000 (Thermo Fischer Scientific, USA) with an RS binary pump and Thermo Scientific Q Executive Orbitrap with Kinetex 2.6 µm EVO C18 (150 x 3.0 mm) reversed-phase column from Phenomenex (Torrence, CA, USA).

For LC separation 0.1% formic acid (Merck, Darmstadt Germany) solution and acetonitrile (HPLC grade, Riedel-de-Haën, Seelze Germany) were used. The ultrapure water with resistance 18.2 MΩ cm and TOC < 5 ppb was prepared by Milli-Q IQ 7000 device from Merck (Darmstadt, Germany). The gradient started with 5% acetonitrile and was increased to 100% over 20 min, was kept at 100% acetonitrile for 5 min, and then lowered back to 5% over 0.1 min. The system was equilibrated for 5 min between runs. The column oven temperature was 40 ˚C and the mobile phase flow rate was 0.350 mL/min.

The autosampler temperature was kept at 15 ˚C. Auxiliary gas, sheath gas, and sweep gas flow rates were set to 3, 35, and 0 arbitrary units, respectively. The auxiliary gas and capillary temperature were 320 ˚C and the S-lens RF level was 50%. For MS^2 data ramp was used in collision energy.

An inclusion list was used to trigger MS^2 spectra for chemicals spiked to the samples. All together three solutions were analyzed containing compounds shown in Table S4 to Table S6. However, out of 152 unique spiked chemicals, 90 were in final calculations and all chemicals were not detected with MS^2 spectra when using $[M+H]^+$ and $[M-H]^-$ as precursors.

Table S4. The first solution used for spiking water samples for application of MS2Tox containing 89 chemicals. Chemicals detected with LC-HRMS/MS are assigned as Minc270001-Minc270064 in application results file https://github.com/kruvelab/MS2Tox/blob/main/Data/Application_results_with_MS2Tox.csv.

**Compound name**	**Monoisotopic mass (Da)**	**Concent-ration (µmol/L)**	**DTXSID from CompTox**	**Detected with MS²**
L-lysine	146.188	10.91	DTXSID6023232	-
Imidazole	68.077	23.63	DTXSID2029616	-
Creatinine	113.118	14.70	DTXSID8045987	-
Pyridine	79.100	19.75	DTXSID9021924	-
Citrulline	175.186	9.21	DTXSID80883373	-
Butylamine	73.137	22.93	DTXSID1021904	-
Tyramine	137.179	12.08	DTXSID2043874	-
Aniline	93.126	17.51	DTXSID8020090	-
Cotinine	176.215	9.17	DTXSID1047576	-
Nicotinamide	122.125	12.84	DTXSID2020929	-
Benzylamine	107.153	14.6	DTXSID5021839	-
Acephate	183.166	7.97	DTXSID8023846	+
Phenylethylamine	121.180	12.38	DTXSID5058773	-
Trimethoprim	290.318	5.08	DTXSID3023712	+
Gabapentin	171.237	9.11	DTXSID0020074	-
O-Desmethylvenlafaxine	263.375	5.83	DTXSID40869118	+
2,2-Dipyridyl	156.184	9.41	DTXSID9040635	-
4-Chloroaniline	127.572	13.03	DTXSID9020295	-
Theophylline	180.164	9.17	DTXSID5021336	+
Sulfapyridine	249.289	6.39	DTXSID3026067	+
Propylthiouracil	170.232	9.10	DTXSID5021209	-
Methomyl	162.210	9.84	DTXSID1022267	+
Caffeine	194.191	8.32	DTXSID0020232	+
Monocrotophos	223.164	7.59	DTXSID9034816	+
1H-Benzotriazole	119.124	12.05	DTXSID6020147	+
Sulfamethazine	278.330	5.65	DTXSID6021290	+
Atraton	211.264	7.20	DTXSID0037493	+
Phenazone	188.226	7.91	DTXSID6021117	+
Fluconazole	306.271	4.86	DTXSID3020627	+
Quinoxaline	130.147	11.22	DTXSID6049432	-
Imidacloprid	255.661	8.17	DTXSID5032442	+

Chloridazon	221.643	7.26	DTXSID3034872	+
5-Methyl-1H-benzotriazole	133.151	11.92	DTXSID1038743	+
Chlorthalidone	338.766	4.79	DTXSID4022812	+
Haloperidol	375.864	4.53	DTXSID4034150	+
Triethyl_phosphate	182.155	9.41	DTXSID8026228	+
Imazalil	297.180	5.00	DTXSID8024151	+
Ametryn	227.330	6.96	DTXSID1023869	+
Ketoconazole	531.431	2.88	DTXSID7029879	+
Climbazole	292.761	5.37	DTXSID6046555	+
Benzothiazole	135.186	12.87	DTXSID7024586	-
Reserpine	608.679	2.67	DTXSID7021237	+
Simazine	201.657	8.13	DTXSID4021268	+
Monuron	198.649	7.37	DTXSID0020311	+
Clotrimazole	344.837	4.21	DTXSID7029871	+
Cyanazine	240.693	6.37	DTXSID1023990	+
Prometryn	241.356	6.51	DTXSID4024272	+
Carbamazepine	236.269	6.98	DTXSID4022731	+
Metsulfuron-methyl	381.364	4.16	DTXSID6023864	+
Irbesartan	428.529	3.61	DTXSID0023169	+
Irgarol	253.367	6.25	DTXSID3032416	+
Corticosterone	346.460	4.38	DTXSID6022474	+
Atrazine	215.683	6.88	DTXSID9020112	+
1-Nitropyrene	247.248	6.19	DTXSID6020983	-
Diuron	233.095	6.92	DTXSID0020446	+
Hydrocortisonacetate	404.497	3.78	DTXSID0048686	+
9,10-Phenanthrenequinone	208.212	7.28	DTXSID3058901	+
Metazachlor	277.749	5.84	DTXSID4058156	+
Norethindrone	298.419	5.31	DTXSID9023380	+
2-(Methylthio)benzothiazole	181.278	8.94	DTXSID70274236	+
Valsartan	435.519	3.35	DTXSID6023735	+
Sebuthylazine	229.710	6.68	DTXSID7058171	+
Ethoprop	242.339	6.95	DTXSID4032611	+
Iminostilbene	193.244	7.93	DTXSID6040458	-
Metolachlor	283.794	5.47	DTXSID4022448	+
Alachlor	269.767	6.04	DTXSID1022265	+
1-hydroxypyrene	218.250	7.47	DTXSID1038298	-
Glimepiride	490.616	3.10	DTXSID5040675	+

Ethyl_azinphos	345.378	4.45	DTXSID5037498	+
Efavirenz	315.675	4.84	DTXSID9046029	+
Progesterone	314.462	5.03	DTXSID3022370	+
Parathion	291.261	5.30	DTXSID7021100	-
Tri-isobutyl-phosphate	266.314	5.87	DTXSID8040698	+
Carbazole	167.207	8.88	DTXSID4020248	-
Triphenylphosphate	326.283	5.06	DTXSID1021952	+
Simvastatin	418.566	3.69	DTXSID0023581	+
Sudan_I	248.279	6.60	DTXSID4021135	+
Chlorpyrifos	350.586	4.27	DTXSID4020458	-
Cyclamic_acid	179.237	8.08	DTXSID5041809	+
Chloramphenicol	323.129	4.71	DTXSID7020265	+
Furosemide	330.744	4.65	DTXSID6020648	+
Dicamba	221.037	6.94	DTXSID4024018	-
1-Naphthol	144.170	10.33	DTXSID6021793	-
Bicalutamide	430.373	3.87	DTXSID2022678	+
Fipronil	437.148	3.60	DTXSID4034609	-
Gemfibrozil	250.333	5.83	DTXSID0020652	-
Linoleic_acid	280.445	9.69	DTXSID2025505	-
4-Amino-6-chloro-1,3-benzenedisulfonamide	285.728	5.20	DTXSID1059521	-
Propanil	218.080	7.22	DTXSID8022111	+

Table S5. The second solution used for spiking water samples for application of MS2Tox containing 28 chemicals. Chemicals detected with LC-HRMS/MS are assigned as Einc270002-Einc270019 in application results file https://github.com/kruvelab/MS2Tox/blob/main/Data/Application_results_with_MS2Tox.csv.

Compound name	Monoisotopic mass (Da)	Concent-ration (µmol/L)	DTXSID from CompTox	Detected with MS^2
Imidazole	68.077	17.34	DTXSID2029616	-
2,6-Diaminopyridin	109.129	8.92	DTXSID0040127	-
Benzylamine	107.153	9.78	DTXSID5021839	-
Acridine	179.217	6.05	DTXSID8059766	-
Ampicillin	349.405	2.05	DTXSID9020083	-
Danofloxacin	357.379	3.23	DTXSID0046432	+
Quinine	324.417	3.84	DTXSID0044280	+
Thiabendazole	201.248	5.52	DTXSID0021337	+
Ofloxacin	361.368	2.20	DTXSID3041085	+
Dazomet	162.276	8.06	DTXSID7024902	-
Methomyl	162.210	5.59	DTXSID1022267	+
Mianserin	264.365	3.14	DTXSID6023317	+
Cyanazine	240.693	3.31	DTXSID1023990	+
dimethyl phthalate	194.184	7.16	DTXSID3022455	+
5,5-Diphenylhydantoin	251.260	3.58	DTXSID8020541	-
(S)-3-Anilino-5-methyl-5-phenylimidazolidine-2,4-dione	281.309	3.84	DTXSID10583591	-
Miconazole nitrate	479.141	1.85	DTXSID50996767	-
Matalaxyl	279.332	2.79	DTXSID6024175	-
Ketoprofen	254.281	3.67	DTXSID6020771	+
Naproxen	230.259	4.93	DTXSID4040686	-
Propanil	218.080	3.77	DTXSID8022111	+
Methidathion	302.331	4.43	DTXSID5020819	-
Diclofenac Sodium	295.141	3.67	DTXSID3037208	-
Febantel	446.477	1.88	DTXSID6046898	+
Anziphos-ethyl	345.378	3.80	DTXSID5037498	+
Tetramethylthioram disulphide	240.433	4.47	DTXSID5021332	-

Diazinon	304.346	3.23	DTXSID9020407	+
diphenyl phthalate	318.323	3.06	DTXSID3021778	+

Table S6. The third solution used for spiking water samples for application of MS2Tox containing 64 chemicals. Chemicals detected with LC-HRMS/MS are assigned as SBinc270001-SBinc270048 in application results file https://github.com/kruvelab/MS2Tox/blob/main/Data/Application_results_with_MS2Tox.csv.

Compound name	Monoisotopic mass (Da)	Concent-ration (µmol/L)	DTXSID from CompTox	Detected with MS2
Histamine	111.08	1.18	DTXSID4023125	+
Guanylurea	102.054	$7.22*10^1$	DTXSID3043811	+
Metformin	129.101	$1.22*10^1$	DTXSID2023270	+
Amitrole	84.044	1.20	DTXSID0020076	+
Atrazine-desethyl-desisopropyl-2-hydroxy	127.049	$1.75*10^1$	DTXSID3060950	-
Butylamine	73.089	$2.24*10^1$	DTXSID1021904	-
Atrazine-desisopropyl-2-hydroxy	155.081	$8.82*10^2$	DTXSID6074756	+
Atrazine-desethyl-2-hydroxy	169.096	$6.32*10^2$	DTXSID80173802	+
Adenosine	267.097	$9.26*10^2$	DTXSID1022558	+
Methamidophos	141.001	1.47	DTXSID6024177	-
Atrazine-desethyl-desisopropyl	145.016	$7.84*10^2$	DTXSID1037806	-
2-Aminobenzothiazole	150.025	$3.8 *10^3$	DTXSID1024467	+
Vancomycin	1447.43	$3.92*10^2$	DTXSID0042664	-
Omethoate	213.022	$4.98*10^2$	DTXSID4037580	+
Atrazine-2-hydroxy	197.128	$2.08*10^2$	DTXSID6037807	+
Chlorothiazide	294.949	$4.56*10^1$	DTXSID0022800	+
Benzotriazole-5-carboxylic acid	163.038	$4.24*10^1$	DTXSID10881049	+
Caffeine	194.080	$8.10*10^1$	DTXSID0020232	+
Caffeine-13C3	196.098	$1.68*10^1$	DTXSID20437172	-
Atrazine-desisopropyl	173.047	$3.44*10^1$	DTXSID0037495	-
Benzotriazole	119.048	1.46	DTXSID6020147	+
Trichlorfon	255.923	$3.67*10^1$	DTXSID0021389	-
Atrazine-desethyl	187.062	$4.28*10^2$	DTXSID5037494	+
5-Methyl-1H-benzotriazole	133.064	$6.42*10^2$	DTXSID1038743	+
Haloperidol	375.14	$1.90*10^2$	DTXSID4034150	+

10.11-Dihydro-10-hydroxycarbamazepine	254.106	$3.26*10^2$	DTXSID50865484	-
5-Chlorobenzotriazole	153.009	$1.74*10^1$	DTXSID0047450	+
Butocarboxim	190.078	$6.42*10^1$	DTXSID5058062	-
Imazalil-d5	301.072	$1.55*10^1$	-	+
Imazalil	296.048	$3.85*10^1$	DTXSID8024151	+
Tylosin	915.519	$1.26*10^1$	DTXSID3043996	-
Carbamazepine-10,11-epoxide	252.270	$2.28*10^2$	DTXSID60891456	+
Ketoconazole	530.149	$1.30*10^1$	DTXSID7029879	+
Climbazole	292.098	$1.48*10^2$	DTXSID6046555	+
Benzothiazole	135.014	$9.58*10^1$	DTXSID7024586	-
Clarithromycin	747.477	$1.66*10^1$	DTXSID3022829	-
Simazine	201.078	$5.54*10^2$	DTXSID4021268	+
Clotrimazole	344.108	$3.74*10^2$	DTXSID7029871	+
Reserpine	608.273	$1.58*10^2$	DTXSID7021237	+
Dichlorvos	219.946	$7.96*10^1$	DTXSID5020449	-
Carbamazepine	236.095	$4.22*10^1$	DTXSID4022731	+
2-Methylbenzothaizole	181.002	$1.51*10^1$	DTXSID8049208	-
Irgarol	253.136	$3.19*10^1$	DTXSID3032416	+
Atrazine-d5	202.159	$2.08*10^1$	DTXSID20486781	-
Atrazine	215.094	$4.75*10^1$	DTXSID9020112	+
Phenazine	180.069	$2.52*10^2$	DTXSID2059069	+
Spinosad	731.461	$1.07*10^1$	DTXSID7032478	+
Metazachlor	277.098	$3.20*10^2$	DTXSID4058156	+
Emamectin	885.524	$1.21*10^1$	DTXSID0034566	-
2-(Methylthio)benzothiazole	181.002	$8.28*10^2$	DTXSID70274236	+
TCMTB	237.969	$5.12*10^1$	DTXSID6032647	+
Metolachlor-OXA	279.147	$2.98*10^1$	DTXSID6037568	+
Rifaximin	785.352	$1.45*10^1$	DTXSID7045998	+
Metolachlor	283.134	$8.38*10^1$	DTXSID4022448	+
Efavirenz	315.027	$3,14*10^1$	DTXSID9046029	+
Progesterone	314.225	$3.42*10^2$	DTXSID3022370	+
Metolachlor-ESA	329.13	$3.08*10^1$	DTXSID1037567	+
Simvastatin	418.272	$2.77*10^1$	DTXSID0023581	+
Sudan I	248.095	$2.14*10^2$	DTXSID4021135	+
Chlorpyrifos	348.926	$6.10*10^1$	DTXSID4020458	-
Octocrylene	361.204	$3.07*10^1$	DTXSID9025299	-
Sucralose	396.015	$4.04*10^1$	DTXSID1040245	-

2-Hydroxybenzothiazole	151.009	$7.90*10^2$	DTXSID6061315	-
Nigericin	724.476	$1.60*10^1$	DTXSID9041079	-

Fingerprints calculated from the structure

The toxicity prediction model was trained to predict LC_{50} and EC_{50} values from chemical fingerprints. For training purposes the fingerprints were calculated from the SMILES of the chemical with *get.fingerprint()* function in R package *rcdk*. Four different types of fingerprints, altogether 1263, were calculated: substructure, MACCS, PubChem and Klekota-Roth. These fingerprint types were used as these can also be calculated by SIRIUS+CSI:FingerID. Fingerprint list with descriptions can be found in a file https://github.com/kruvelab/MS2Tox/blob/main/Data/final_fingerprints_SI.txt in GitHub. In this file there are six columns:

absoluteIndex – unique number given to each fingerprint,

description – showing fingerprint SMARTS,

rowpos – row number from SIRIUS calculated fingerprint list,

rowneg – row number from SIRIUS calculated fingerprint list,

fp_type – fingerprint group,

colname – unique name given for fingerprint-based on SIRIUS absoluteIndex which is used as column name later in building the prediction model.

In addition to fingerprints, the exact mass of the compound was used as a descriptive feature.

We additionally evaluated the overlap of the training, test, and validation set in the chemical space. For this the substructure, MACCS, PubChem, and Klekota-Roth fingerprints were calculated with *rcdk* for all chemicals in the three sets and used as an input to the principal component analysis (PCA). The first two principal components shown in Figure S2 explained 16 % of the total variability in the fingerprints. In spite of the low explained variability, the visual analysis of the first two principal components assured a good overlap in the chemical space of the training, test, and validation set, see Figure S2.

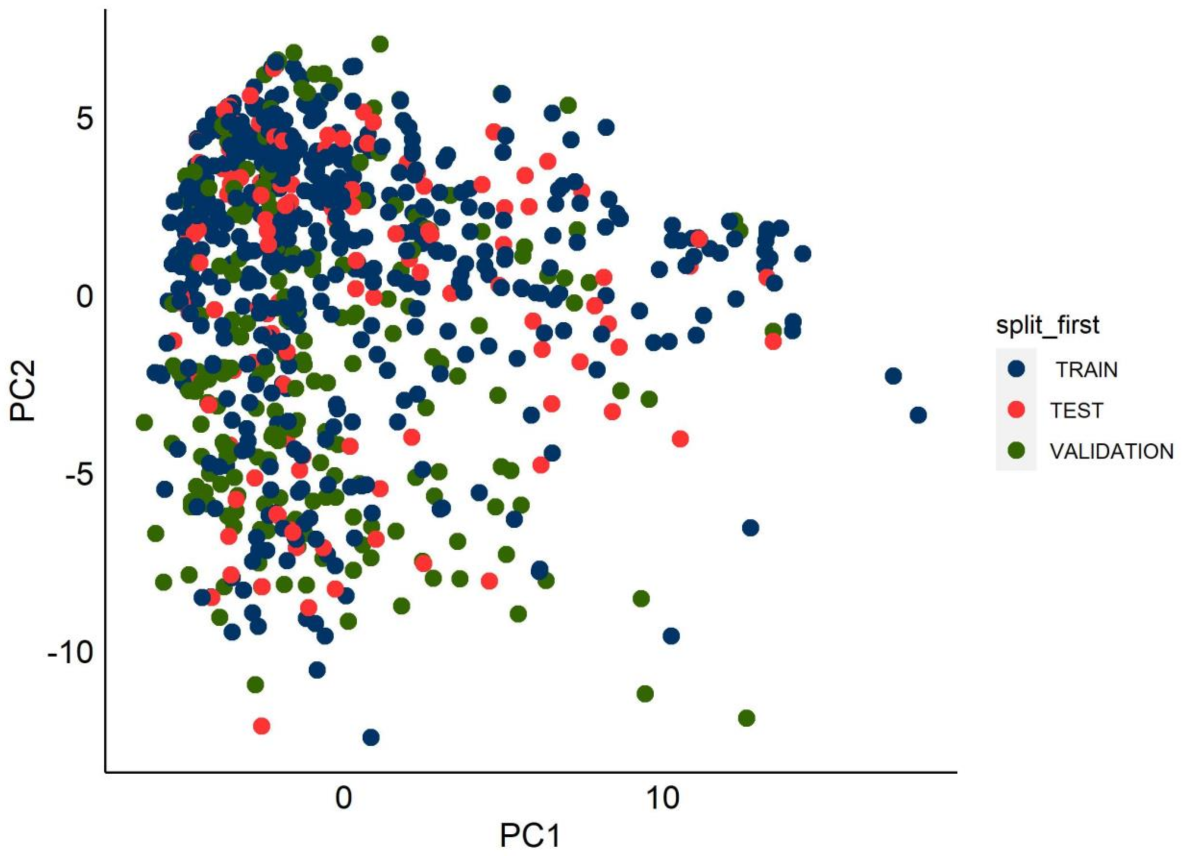

Figure S2. PC1 and PC2 graph for static fish LC_{50} dataset. Different colors show the training, test, and validation set.

Data purification before model training

Before training near-zero variance fingerprints and highly correlated fingerprints were filtered out using *caret* package functions *nearZeroVar()* with default parameters and *findCorrelation()* and *cor()* with cutoff = 0.8. The number of remaining fingerprints varied from 191 to 243 depending on the species and endpoint and is shown in Table S6. The final lists of fingerprints are shown in files

https://github.com/kruvelab/MS2Tox/blob/main/Data/TrainTest/xgbDART_fish_stat_testtrain_results.csv,

https://github.com/kruvelab/MS2Tox/blob/main/Data/TrainTest/xgbDART_fish_flow_testtrain_results.csv,

https://github.com/kruvelab/MS2Tox/blob/main/Data/TrainTest/xgbDART_LC50_waterflea_testtrain_results.csv,

https://github.com/kruvelab/MS2Tox/blob/main/Data/TrainTest/xgbDART_EC50_waterflea_testtrain_results.csv,

https://github.com/kruvelab/MS2Tox/blob/main/Data/TrainTest/xgbDART_EC50_algae_testtrain_results.csv for each model in GitHub.

Table S7. Final number of fingerprints used for model building

Model	Fingerprint amount
Fish Stat LC_{50}	243
Fish Flow LC_{50}	203
Water flea LC_{50}	192
Water flea EC_{50}	191
Algae EC_{50}	206

Fingerprints calculated with SIRIUS software

For toxicity predictions for mass-spectrometric data, fingerprints were calculated from HRMS data using SIRIUS+CSI:FingerID[2] application. SIRIUS+CSI:FingerID computes the fingerprints for plausible molecular formulas from .ms files containing MS^1 and MS^2 spectra. SIRIUS+CSI:FingerID constructs a fragmentation tree where smaller fragments are related to the higher fragments in a consecutive manner. In this construction, the fragments and losses with plausible molecular formulas are organized relative to each other. Fingerprints are then predicted using these constructed fragmentation trees. In the process of creating a fragmentation tree also different plausible molecular formulas are created, within the allowed mass accuracy. However, MS2Tox does not rely on the molecular formula assignment, only on the predicted fingerprints. Therefore, in the context of MS2Tox, the molecular formula predicted by SIRIUS is a by-product. To study how different are fingerprints from the same MS2 data but with different assigned formulas, cosine similarities were calculated for each data point. The histogram in Figure S3 shows that for most of the chemicals, fingerprints were very similar with average cosine similarities over 0.6 and mostly over 0.8 even. These results prove that fingerprints calculated from the same MS2 are similar even with the wrong assigned formula and thus can give the correct predicted toxicity value.

For many HRMS spectra several (occasionally up to 11) possible chemical formulas were predicted. From 680 spectra 306 had more than one possible formula calculated by SIRIUS+CSI:FingerID. 39 spectra had more than ten or more possible molecular formulas. From 680 results correct formula was ranked among three best-ranked formulas on 674 times and the best rank 592 times, meaning that 13% of the cases incorrect formula was rank one. For the fish static model in 8% of the cases, an incorrect formula was ranked highest. In spite of the occasional highest ranking of incorrect formulas, for final toxicity predictions, only the first rank is used. In order to find out if this decision was justified, predicted toxicities were also calculated using all formulas and the first three ranked formulas and averaged. Figure 2C in the main text shows that in all three cases the RMSE were comparable and using rank one

formulas gave the most similar results to when using the correct formula. This indicates that even if the correct formula is not ranked highest, the fingerprints can still be predicted accurately.

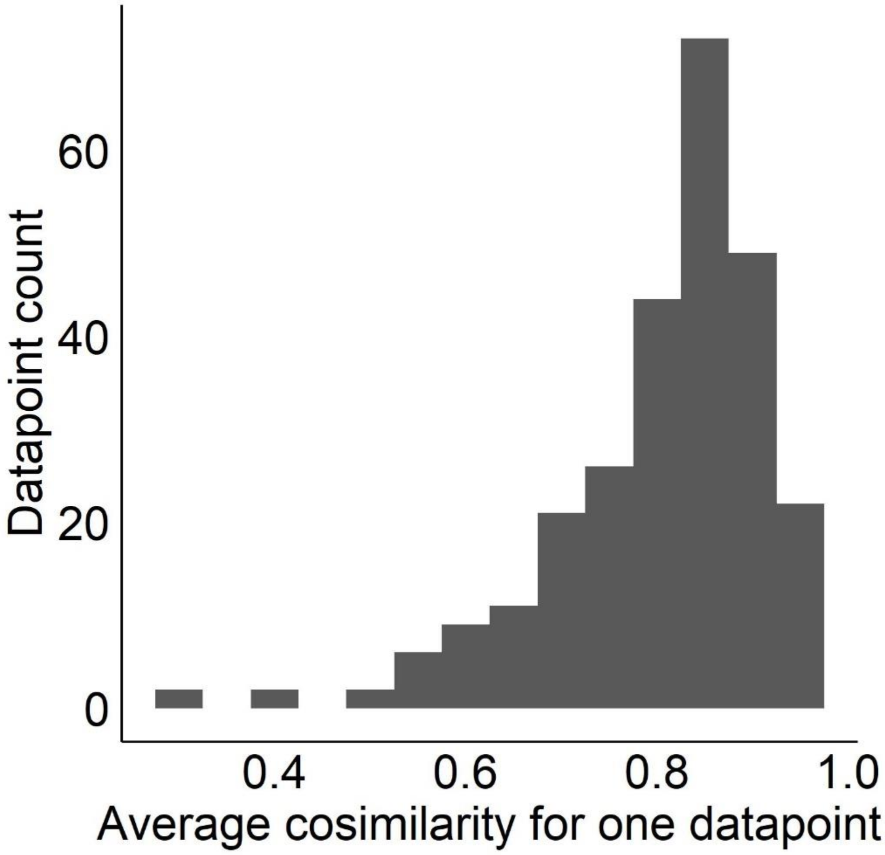

Figure S3. Histogram showing the median cosine similarities between different fingerprints calculated from the same MS2 spectra but with different assigned formulas.

In this work SIRIUS calculations were done using SIRIUS graphical user interface (GUI) version 4.9.5. For fingerprint calculations ".ms" file from each experiment was created so that file contained the compound id, parent mass, MS^2 spectra on different collision energies, and MS^1 data. For MassBank MS^1 spectra were not provided, therefore, an isotope pattern calculated from the correct molecular formula was used. In negative mode additional information about ionization $[M-H]^-$ was provided in the input file as otherwise program assumed usage of positive mode. In positive mode information about ionisation was not added as different adducts like $[M+Na]^+$, $[M+K]^+$ are possible beside $[M+H]^+$. An example of an input file for SIRIUS is shown in GitHub README.

For SIRIUS calculations mass accuracy of 5 ppm for Orbitrap was used and all databases available in SIRIUS+CSI:FingerID software (e.g PubChem, Bio database, NORMAN) were searched for possible structural matches. Some compounds that had a mass over 600 Da could not always be calculated due to too many possible formula matches being found. This

arouses from a too wide range of allowed chemical elements (H, C, N, O, P, B, Si, S, Cl, Se, Br, F, I, K, Na, As).

After SIRIUS+CSI:FingerID calculations a folder that contain all predicted molecular formulas with respective fingerprints, scores, and fragmentation trees is created. Code for gathering this data into a data table can be found in GitHub R package *MS2Tox*.

Performance evaluation for the fingerprint calculation

The fingerprints calculated with SIRIUS+CSI:FingerID for the correct formula were compared to the fingerprints calculated directly from the structure. 98% of the fingerprints were correctly calculated by SIRIUS+CSI:FingerID software. Here correct predictions mean that if a specific fingerprint was present in the structure its predicted probability was above 0.5 as the regression trees treat all values below 0.5 as zeros and above 0.5 as one. However, for some spectra fingerprint calculations were less accurate. In Table S8 are given an overview of the spectra with the most miscalculated fingerprints. As for fingerprint calculations, different collision energies were combined, MassBank ID corresponds to the last file in the list. The number of combined MS^2 on different collision energies is given in column "number of MS^2 spectra". The table is corresponding to fish static data and only those fingerprints are selected that are used in the final models. In Table S9 most miscalculated fingerprints can be seen for the same dataset.

Table S8. Spectral data with most miscalculated fingerprints with SIRIUS+CSI:FingerID for fish static model.

**MassBank ID**	**Number of MS^2 spectra**	**Compound name**	***m/z* range of peaks used by SIRIUS**	**Number of MS^2 peaks used by SIRIUS**	**Nr of miscalculated fingerprints (from 243)**
AU257306	6	Fenpropimorph	57-304	22	78
EA014614	14	Fenpropimorph	55-3014	31	78
LU099856	6	Dicyclohexyl phthalate	57-329	42	76
LU116106	6	Fenoxycarb	53-302	6	76
LU066656	6	Aminocarb	92-209	9	69
LU104606	6	Phenobarbital	53-233	17	69
EQ362909	9	delta9-Tetrahydrocannabinol	15-315	56	66
AU244906	6	Galaxolide	117-259	18	65
LU037956	6	tau-Fluvalinate	232-501	3	64
LU074756	7	Cyhalofop-butyl	65-356	47	63

Table S9. Overview of fingerprints most often miscalculated by SIRIUS+CSI:FingerID for fish static model

**Fingerprints (corresponding to SIRIUS absoluteIndex)**	**Description of the fingerprint taken from SIRIUS+CSI:FingerID software. Fingerprint descriptions are the parts coloured with red.**	**No of chemical with miscalculated fingerprint (from 219 compounds)**
Un1409	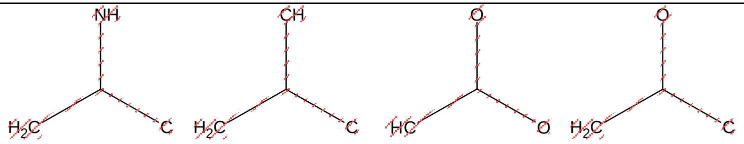	77
Un142	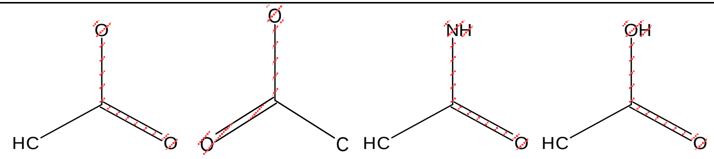	66
Un505	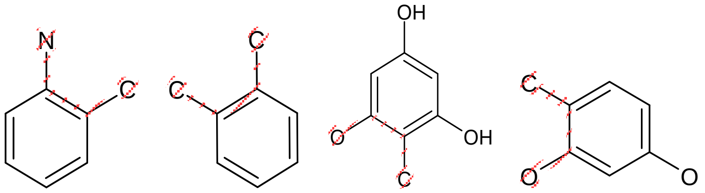	66
Un354	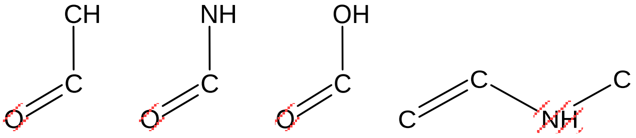	65
Un511	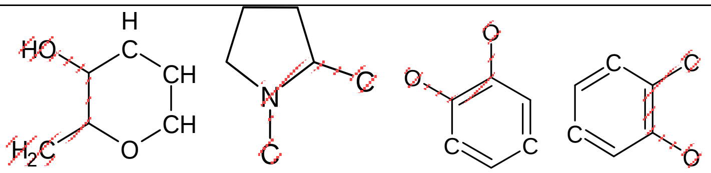	65
Un1171	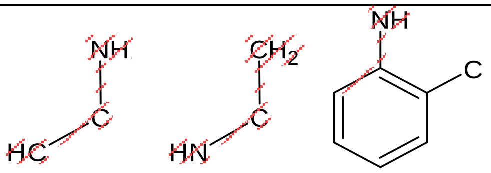	63
Un341	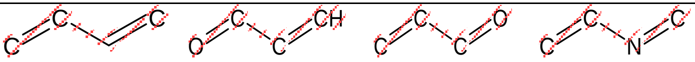	61
Un435	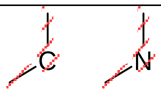	61
Un492	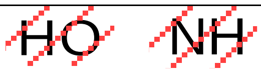	61
Un329	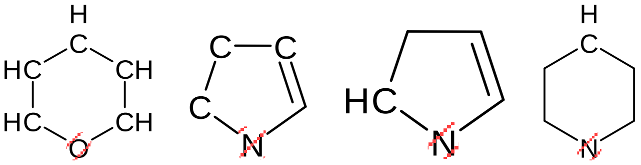	60

Prediction model

For toxicity predictions, an extreme gradient boosted Dropouts Multiple Additive Regression Trees model *xgbDART* was trained with R package *caret*. Due to variability in the repeatability of the endpoint values, each toxicity value had a different weight in the model. Compounds for which the experimental repeatability was poorer had lower weight in the model training.

For calculating weights experimental SD for each chemical was used. For compounds that had several replica measurements weight = 1/SD. For those compounds that did not have replicas, SD was taken as equal to median SD errors over the dataset and then again weight = $1/SD_{median}$. As the range for these weights was really wide (e.g 0.68 to 7459 for fish static) all the weight values were scaled using the logarithmic value of the weight to range 1-5. Conventional scaling strategies would press the weight of less accurate measurements to zero, this was undesirable and the scaling to 1-5 was used instead. Formula to get final weights in scale 1-5: Weight final = $((weight-weight_{min})/(weight_{max}-weight_{min}))*4+1$.

In the case of the three fish model, the LC_{50} values of bluegill and rainbow trout were calculated into fathead minnow scale using the generalized additive model (GAM). Due to this conversion to LC_{50} values of fathead minnow have a higher weight in the model training. For this, initial weights were calculated similarly to the previous: weights = 1/SD, but after that fathead minnow weights were multiplied with two. After that, the weights were scaled to range 1-5.

All chemicals that had HRMS spectra in MassBank were removed from the training set and kept for validation of the models. The remaining chemicals were split 80/20 in training and test set. For final model available in GitHub all three datasets were combined and all compounds were used for training the model.

Comparison of different machine learning models

Five different machine learning approaches were tested for toxicity predictions models. Three of them were different extreme gradient boost, one support vector machine, and one random forest. All other parameters, including selected fingerprints, separation of training and test set, weights and model training parameters, were identical. For training ten-fold cross-validation with “boot” method was used from *caret*. The performance of all used methods is described in Table S10.

Table S10. Comparison of different models, names given according to R package, using fish LC_{50} dataset with static mode and caret package in R. Root mean square error (RMSE) is in log-mM units – lower number shows better results. R^2 shows the correlation – the closer the number is to 1, the better.

**Method (fish, static)**	**RMSE Training**	**RMSE Test**	**R^2 Training**	**Q^2 Test**
xgbTree	0.40	0.85	0.96	0.77
xgbLinear	0.26	0.85	0.98	0.76
**xgbDART**	**0.52**	**0.79**	**0.93**	**0.79**
svmLinear	0.64	1.17	0.90	0.66
rpart	1.17	1.16	0.62	0.48

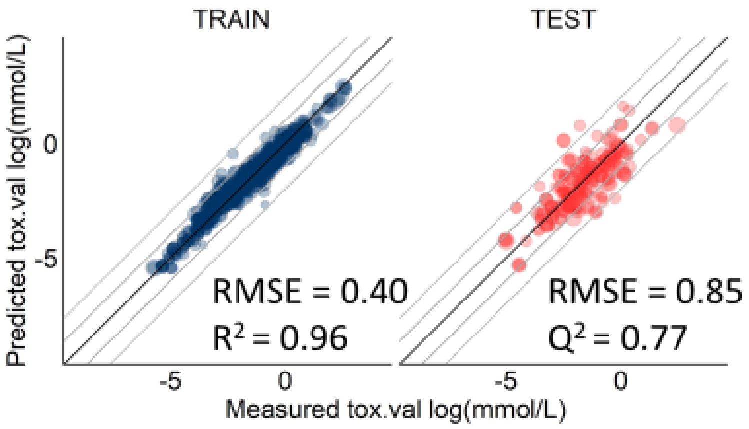

Figure S4. Predicted and measured fish static LC_{50} correlations trained with extreme gradient boosting model xgbTree.

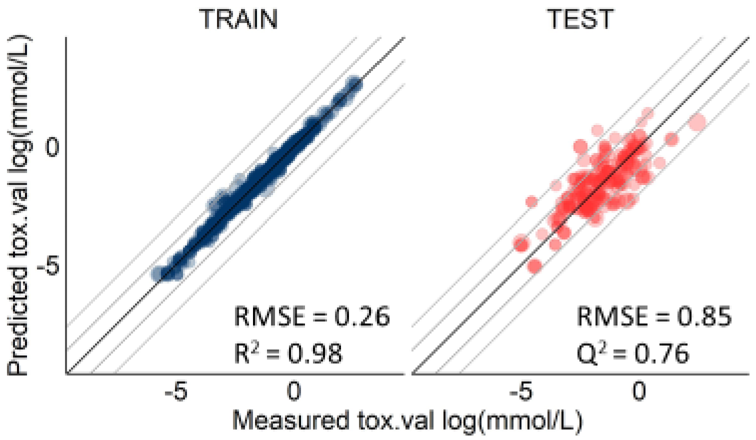

Figure S5. Predicted and measured fish static LC_{50} correlations trained with extreme gradient boosting model xgbLinear.

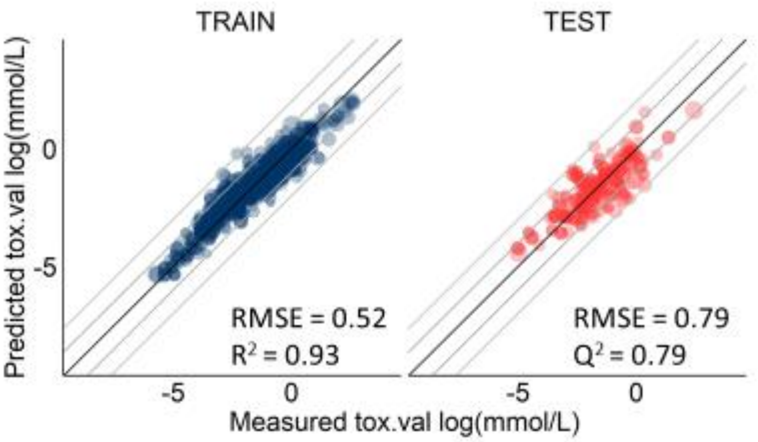

Figure S6. Predicted and measured fish static LC_{50} correlations trained with extreme gradient boosting model xgbDART.

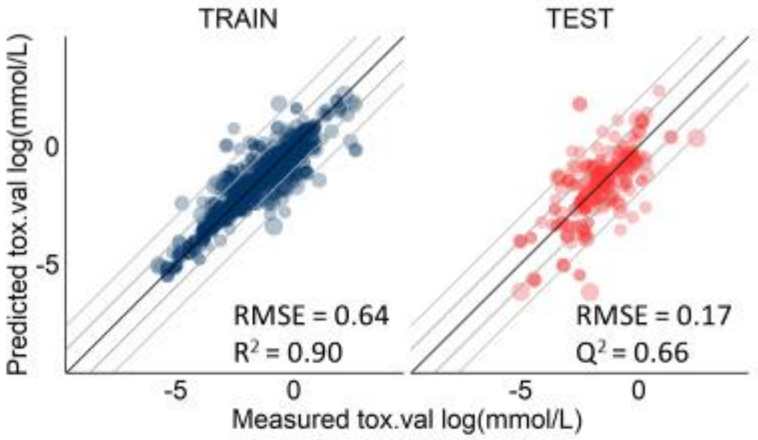

Figure S7. Predicted and measured fish static LC_{50} correlations trained with linear support vector machine model svmLinear.

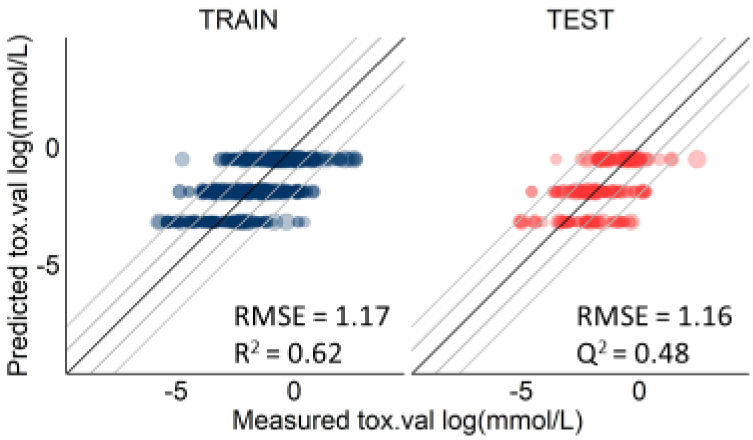

Figure S8. Predicted and measured fish static LC_{50} correlations trained with random forest model rpart. Here random forest only predicts three different toxicity values, which appear as horizontal lines. This may occur if no significant pattern in the data is found or too few data points are available.

Validation with MS^2 data from MassBank

For validation of the method, fingerprints calculated from MS^1 and MS^2 spectra with SIRIUS+CSI:FingerID were used. All compounds used for validation were excluded from the training and test set. Datasets for validation are described above in section "HRMS data from MassBank". The results of the endpoint predictions for the validation set are in GitHub files (https://github.com/kruvelab/MS2Tox/blob/main/Data/Validation.zip) as well as all fingerprints calculated with SIRIUS+CSI:FingerID for MassBank data with respective SiriusScores.

Variable importance and SHAP analysis

In order to understand the information learned by the xgbDART algorithm, the importance and the contribution of each fingerprint to the prediction was evaluated with SHapley Additive exPlanations (SHAP). SHAP graphs for the other four models are given here in addition to the fish static LC_{50} model in the main article text. The bar chart in up left corner shows the relative (to the first variable) importance of the first ten variables in building the model. SHAP graphs are showing the impact on each of those variables in the order of importance. For fingerprints on x-axis 0 and 1 show correspondingly the absence and presence of the fingerprint. Lower SHAP values indicate lower predicted LC_{50} values (higher toxicity), keeping all other parameters constant. Fingerprint naming Un refers to absoluteIndex numbering system in SIRIUS+CSI:FingerID.

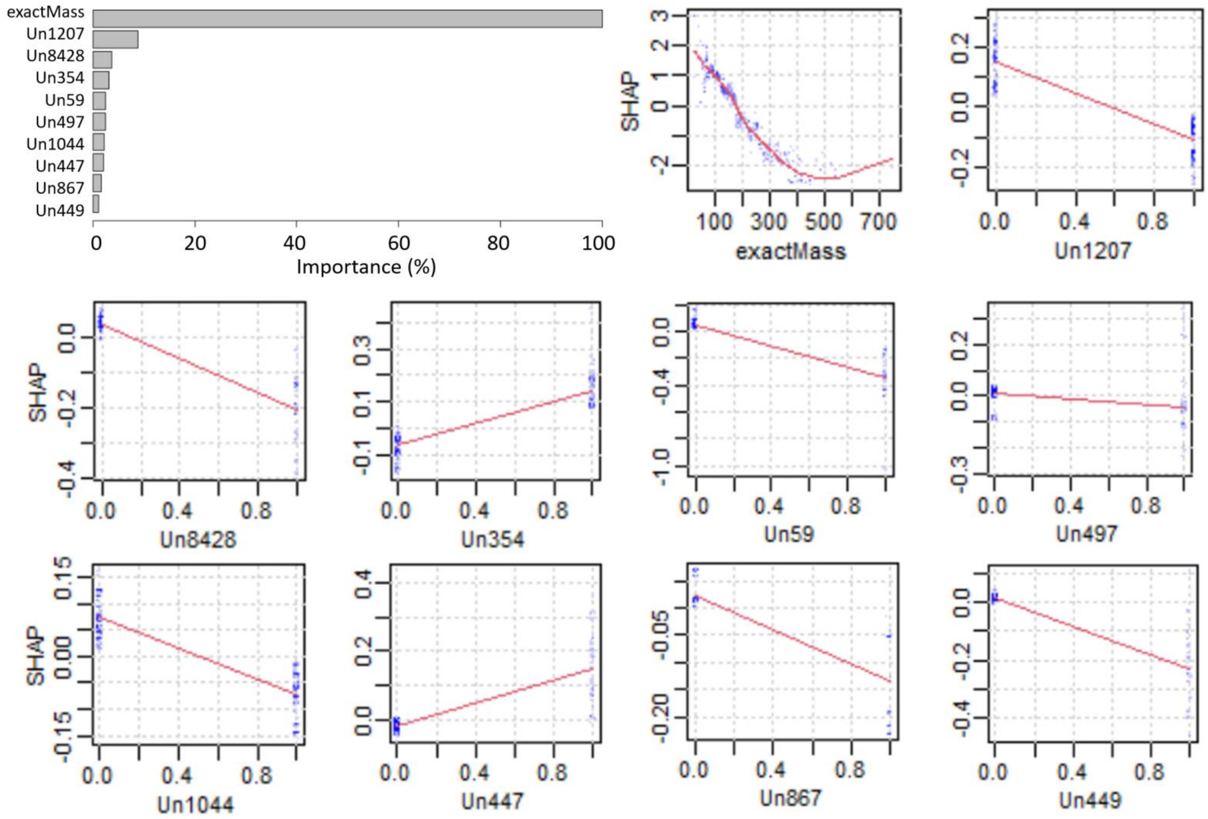

Figure S9. Variable importance analysis for fish flow-through LC_{50} model. Importance and SHAP analysis for first ten variables. The associated SHAP graphs of each variable shows the magnitude and directionality of each variable on the predicted LC_{50}. For each of the molecular fingerprints, the x-axis indicates absence (0) or presence (1) of the respective structural fragment, and lower SHAP values indicate lower predicted LC_{50} values (higher toxicity) assuming all other parameters constant. The line shows the directionality of the impact of the descriptors. Fingerprint naming 'Un' refers to the absolute index numbering system in SIRIUS+CSI:FingerID.

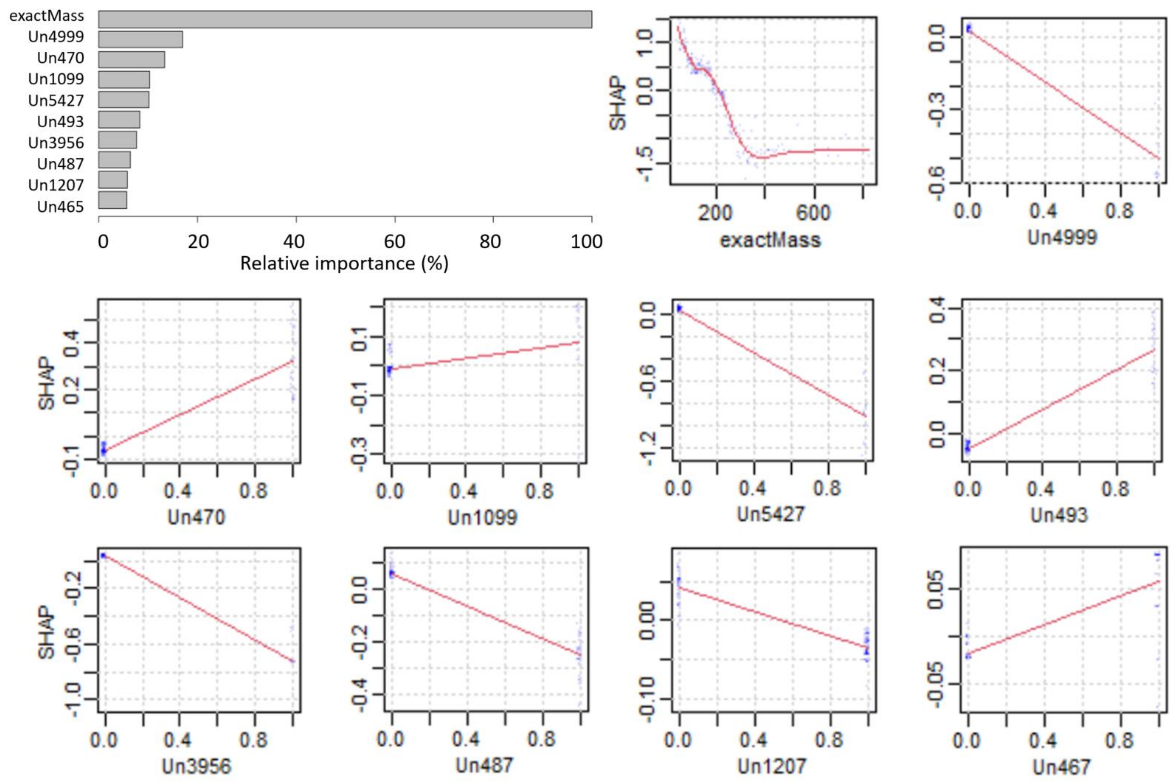

Figure S10. Variable importance analysis for water flea LC_{50} model. Importance and SHAP analysis for first ten variables. The associated SHAP graphs of each variable shows the magnitude and directionality of each variable on the predicted LC_{50}. For each of the molecular fingerprints, the x-axis indicates absence (0) or presence (1) of the respective structural fragment, and lower SHAP values indicate lower predicted LC_{50} values (higher toxicity) assuming all other parameters constant. The line shows the directionality of the impact of the descriptors. Fingerprint naming 'Un' refers to the absolute index numbering system in SIRIUS+CSI:FingerID.

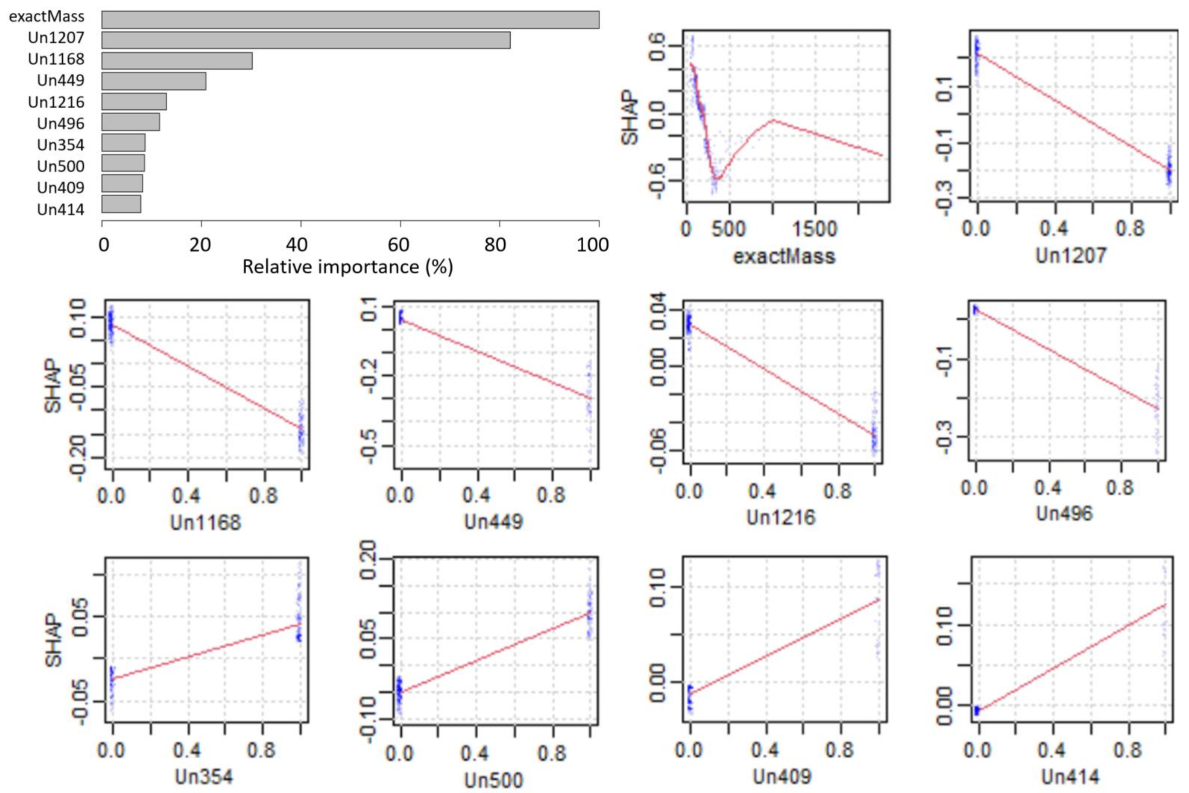

Figure S11. Variable importance analysis for water flea EC_{50} model. Importance and SHAP analysis for first ten variables. The associated SHAP graphs of each variable shows the magnitude and directionality of each variable on the predicted LC_{50}. For each of the molecular fingerprints, the x-axis indicates absence (0) or presence (1) of the respective structural fragment, and lower SHAP values indicate lower predicted LC_{50} values (higher toxicity) assuming all other parameters constant. The line shows the directionality of the impact of the descriptors. Fingerprint naming 'Un' refers to the absolute index numbering system in SIRIUS+CSI:FingerID. The unusual trend line for exactMass is due to the fact that dataset contained one compound having much larger mass than other compounds.

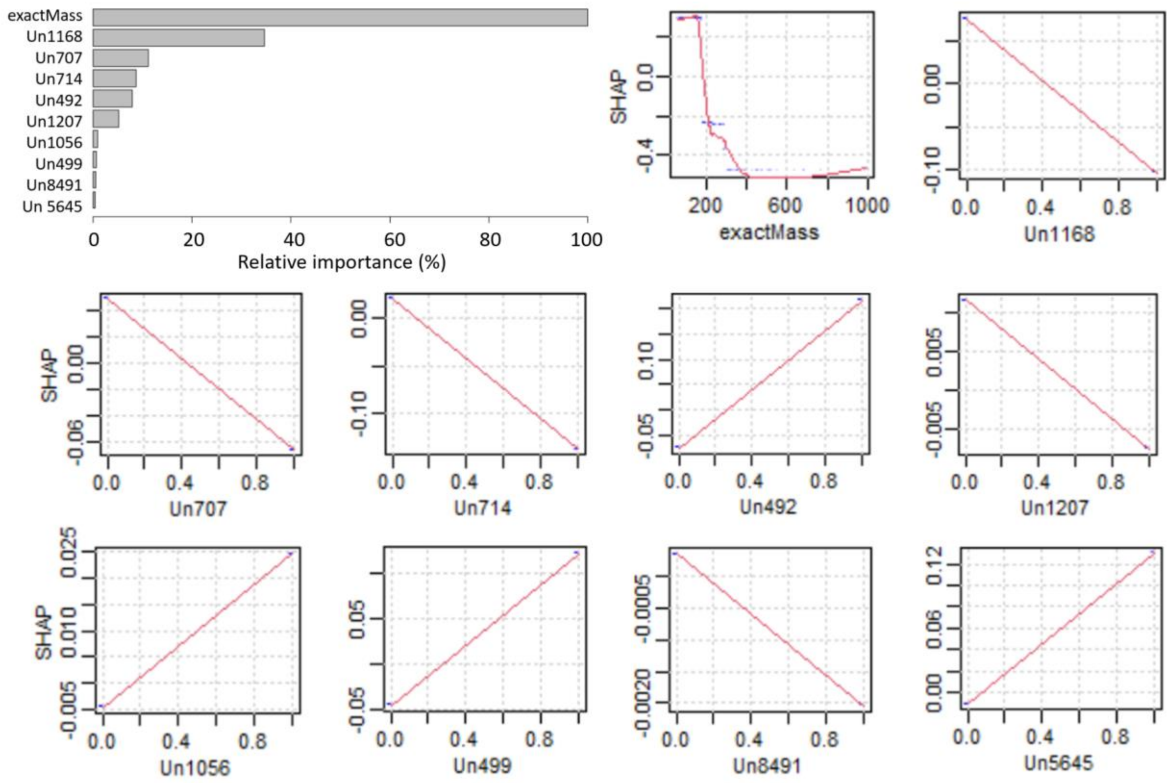

Figure S12. Variable importance analysis for algae EC_{50} model. Importance and SHAP analysis for first ten variables. The associated SHAP graphs of each variable shows the magnitude and directionality of each variable on the predicted LC_{50}. For each of the molecular fingerprints, the x-axis indicates absence (0) or presence (1) of the respective structural fragment, and lower SHAP values indicate lower predicted LC_{50} values (higher toxicity) assuming all other parameters constant. The line shows the directionality of the impact of the descriptors. Fingerprint naming 'Un' refers to the absolute index numbering system in SIRIUS+CSI:FingerID.

Application interpretation

For interpreting spiked solution, rat LD_{50} toxicity values from CompTox were used. Parameters (column names in datasets) for filtrating LD_{50} values:

toxval_units = mg/kg,

toxval_numeric_qualifier = "=",

exposure_route = oral,

risk_assessment_class = acute,

study_type = acute,

study_duration_value = -1.

Predicted toxicity values were compared to measured retention time. It can be seen Figure S13, that there is a negative correlation between retention time and LC_{50} value that indicates that compounds with higher retention time have lower LC_{50}. However, the correlation is vague, meaning that the toxicity predictions by MS2Tox account for also other properties besides the hydrophobicity that is primarily correlated with the retention time. For comparison, experimental fish static LC_{50} were also correlated with MassBank data, see Figure S14. Similarly to Figure S13 only a vague correlation with the retention time is observed, indicating that the mechanism of action also is more complex.

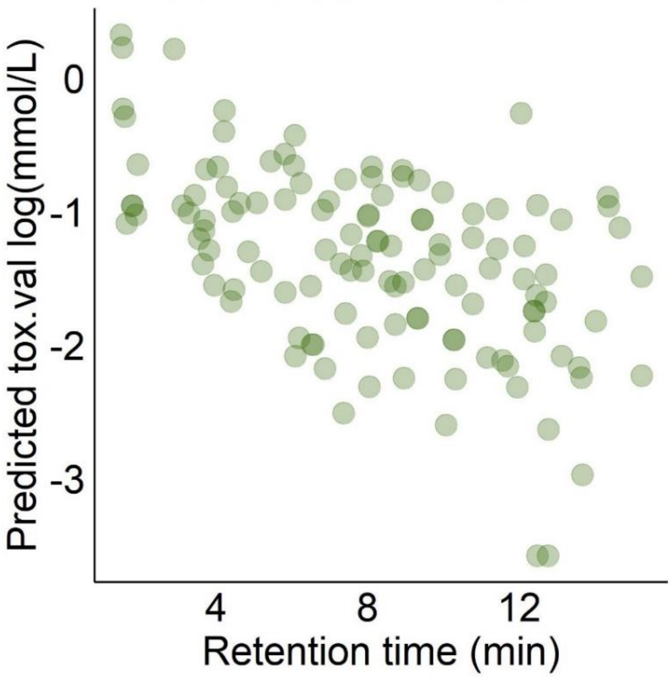

Figure S13. Comparison of measured retention time and predicted toxicity value for spiked application solutions using final fish static LC_{50} model.

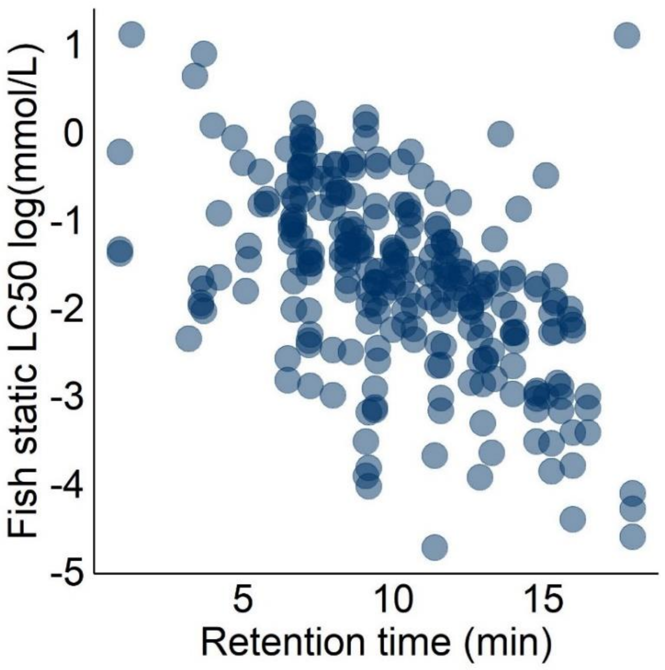

Figure S14. Comparison of measured retention time from Eawag dataset in MassBank and experimental fish static LC_{50} from CompTox.

References

(1) Stanstrup, J.; Neumann, S.; Vrhovšek, U. PredRet: Prediction of Retention Time by Direct Mapping between Multiple Chromatographic Systems. *Anal. Chem.* **2015**, *87* (18), 9421–9428. https://doi.org/10.1021/acs.analchem.5b02287.

(2) Dührkop, K.; Fleischauer, M.; Ludwig, M.; Aksenov, A. A.; Melnik, A. V.; Meusel, M.; Dorrestein, P. C.; Rousu, J.; Böcker, S. SIRIUS 4: A Rapid Tool for Turning Tandem Mass Spectra into Metabolite Structure Information. *Nat Methods* **2019**, *16* (4), 299–302. https://doi.org/10.1038/s41592-019-0344-8.
